# Trends, Drivers and Projections of Pressure Injury Burden in China: Implications for National Healthcare Policy and Aging Governance

**DOI:** 10.1111/iwj.70825

**Published:** 2026-01-25

**Authors:** Ruijuan Li, Xueneng Yang, Jun Shu, Ming Zeng, Junfei Liu, Limin Guo

**Affiliations:** ^1^ Department of Burn The Second Affiliated Hospital of Kunming Medical University Kunming China; ^2^ Department of Traumatic Orthopedics The Second Affiliated Hospital of Kunming Medical University Kunming China; ^3^ Department of Geriatric Orthopedics Affiliated Hospital of Yunnan University Kunming China

**Keywords:** China, global burden of disease, population aging, pressure injuries, trend forecasting

## Abstract

Pressure injuries represent a significant public health challenge in the context of global population aging. As China faces rapid aging, the characteristics of its disease burden and the strategies for prevention and control remain unclear. This study analyses the trends in the disease burden of pressure injuries in China over the past 30 years, based on the Global Burden of Disease (GBD) 2021 data, and predicts the future trends over the next 20 years. The GBD 2021 data were used in combination with the Joinpoint regression model, age‐period‐cohort (APC) model and Bayesian age‐period‐cohort (BAPC) model to analyse the burden of pressure injuries in China from 1990 to 2021 and forecast trends from 2022 to 2040. Additionally, decomposition analysis was performed to quantify the contribution of population aging to the disease burden. In 2021, the number of people with pressure injuries in China was 102 938.9, the number of new cases was 397 312.3, the disability‐adjustede life years (DALYs) totaled 27 383.5 and the number of deaths was 3131.5. The gender difference was characterised by ‘higher prevalence in middle‐aged men, with a reversal in very old women’. Between 1990 and 2021, the burden of pressure injuries significantly increased, with population aging being the main driving factor (46.5%–65.0%). The cohort effect indicated a higher risk for those born before 1942 (RR = 1.04–1.86), and a decreasing risk for those born after 1960 (RR = 0.91). Future projections suggest a 25.8% decrease in total cases, but an increased burden in those aged 85 and older, with deaths rising by 127%. This study highlights the aging‐driven burden of pressure injuries in China, along with gender differences and typical period and cohort effects. As the population ages, the burden of pressure injuries in older age groups will continue to rise. The findings provide evidence for the ‘Healthy China 2030’ initiative and call for the inclusion of pressure injury prevention and control in the core agenda of national aging governance.

## Summary

- First comprehensive national‐level study to assess the long‐term disease burden of pressure ulcers in China using GBD 2021 data and multiple advanced statistical models.
- Significant increase in pressure ulcers burden from 1990 to 2021, with age‐standardized DALY and death rates rising by 117.6% and 100%, respectively, indicating worsening severity despite stable incidence and prevalence trends.
- Population aging identified as the primary driver, accounting for 46.5%–65.0% of the increase in disease burden, especially in the 85+ age group, highlighting a key public health challenge in China's super‐aging transition.
- Marked gender disparities observed: men had higher burden in middle age, while women surpassed men in very old age, reflecting gender‐specific risk patterns and implications for targeted intervention.
- Distinct period and cohort effects revealed by APC models, with peak burden observed in 2009 and among cohorts born before 1942, possibly linked to historical trauma and healthcare access disparities.
- Future projections using the Bayesian APC model predict a 127% increase in deaths by 2040, despite a decline in total prevalence and incidence—indicating a “scissors gap” where burden increasingly concentrates among the elderly.
- Current prevention systems are inadequate for managing pressure ulcers in an aging society; urgent need for policy reform, AI‐enabled community interventions, and elderly‐specific care strategies.
- Proposes establishment of a national “Elderly Pressure Ulcer Prevention Fund” and integration of pressure ulcer management into China's Healthy Aging and Healthy China 2030 strategies.

**Abbreviations**
AAPC: Average annual percentage change
APC: Age‐period‐cohort
BAPC: Bayesian age‐period‐cohort
CI: Confidence interval
DALYs: Disability‐adjusted life years
EAPC: Estimated Annual percentage change
GBD: Global Burden of Disease
ICD: International Statistical Classification of Diseases
UI: Uncertainty interval

## 1 Introduction

Pressure injuries are structural damage to the skin and/or subcutaneous tissue caused by localised mechanical loading. Their development involves external forces, local microenvironmental conditions and individual susceptibility. External pressure exceeding the capillary perfusion threshold leads to tissue ischemia, hypoxia and necrosis [1, 2]. Shear forces cause displacement between tissue layers, distorting blood vessels and connective tissues, resulting in deep damage. Friction damages the stratum corneum, reduces skin tolerance and increases the risk of breakdown. In the local environment, prolonged exposure to moisture—such as sweat, urine or faeces—weakens the skin barrier and contributes to incontinence‐associated dermatitis, further worsening tissue damage. Intrinsic factors influence tissue tolerance and repair, including limited mobility, poor nutrition, advanced age, diabetes and cardiovascular disease [1]. The global incidence of pressure injuries ranges from 6% to 20% [3, 4]. Patients suffer from long‐term pain and discomfort, which significantly reduces their quality of life, while also facing psychological, physiological and social health issues, such as social isolation and body image disturbances caused by wound exudate or odour [5]. Moreover, pressure injuries impose a heavy economic burden. Studies show that the average daily treatment cost for pressure injuries ranges from $1.84 to $150 [6, 7, 8, 9]. In the United States, annual treatment costs can reach up to $26.8 billion [10], and in the Netherlands, the rehabilitation cost per pressure injury patient can be as high as €15 412, creating a significant socioeconomic burden [11].

China, as the most populous country in the world, is facing an increasingly severe disease burden. By 2023, the population aged 60 and above in China reached 297 million, accounting for 21.1% of the total population, with this proportion growing at an annual rate of 3.2% [12]. It is projected that within the next 20 years, China will surpass Japan to become the country with the most severe aging population globally [13]. Meanwhile, the prevalence of diabetes in China has increased from 14.1% in 2016 to 18.8%, showing a continuous upward trend [14, 15]. Additionally, the incidence of obesity and overweight has risen from around 20% to approximately 28% [16, 17]. The increase in chronic diseases, coupled with the intensifying aging population, further highlights the significance of the elderly in Chinese society, necessitating urgent attention [18, 19]. As a common age‐related disease with a prolonged chronic course, pressure injuries have a significant impact in China [20, 21, 22]. Despite some progress in the treatment of pressure injuries, epidemiological research on the condition remains relatively scarce, with most studies focused on individual provinces or single centres, lacking nationwide systematic analysis. As a result, the trend in the disease burden of pressure injuries is not yet clear, and effective future projections are lacking, which hinders the scientific planning of chronic disease management and future prevention efforts in the ‘Healthy China 2030’ initiative [23, 24, 25, 26].

This study used data from the Global Burden of Disease Study 2021 (GBD 2021) to examine the prevalence and trends of pressure injuries among older adults in China over the past three decades. It explored the effects of age, period and birth cohort on the burden of disease and projected future trends through 2040, aiming to support the development of prevention and management strategies in the context of population aging.

## 2 Materials and Methods

### 2.1 Data

To achieve the study objective of analysing the changes in the disease burden of pressure injuries, we selected the GBD 2021 as the data source. To the best of our knowledge, the GBD database is the most comprehensive and widely used public database for pressure injury data. GBD 2021 provides data on the disease burden of 371 diseases across 204 countries and regions globally, and 811 subnational areas. The open‐source data used in this study can be accessed from the GBD 2021 database, which includes the following: [1] incidence, prevalence, death and Disability‐adjusted life years (DALYs) for pressure injuries in China from 1990 to 2021, categorised by gender (overall, male and female), along with their respective age‐standardised rates; [2] global population projections from 2022 to 2040.

### 2.2 Definition

In GBD 2021, pressure injuries are defined as: ‘Decubitus ulcer, also known as pressure ulcer or sore, is an injury to the skin and underlying tissue resulting from an obstruction of blood flow due to pressure on the skin’ (ICD‐10: L89). This study selected incidence, prevalence, death and DALYs as the core indicators for measuring the disease burden of pressure injuries. The study population includes individuals ranging from 0 to 95 years and older, with two key metrics: case numbers and age‐standardised rates. Case numbers represent the raw number of cases recorded in GBD 2021, while age‐standardised rates are the number of cases per 100 000 population, adjusted for age structure, to facilitate more consistent comparisons across regions. All raw data from GBD 2021 are accompanied by 95% uncertainty intervals (UI), which are derived through aggregation and weighted processing.

### 2.3 Statistical Analysis

This study employed the following statistical methods: Joinpoint regression analysis, Age–period–cohort analysis, Decomposition Analysis and BAPC models. All statistical analyses and data visualisations were conducted using R 4.3.1, with statistical significance set at *p* < 0.05. All estimates were based on the original data from the GBD database and included 95% UI. (For detailed methodology, please refer to the Supporting Information.)

## 3 Results

### 3.1 Disease Burden of Pressure Injuries in China in 2021

**FIGURE 1:**
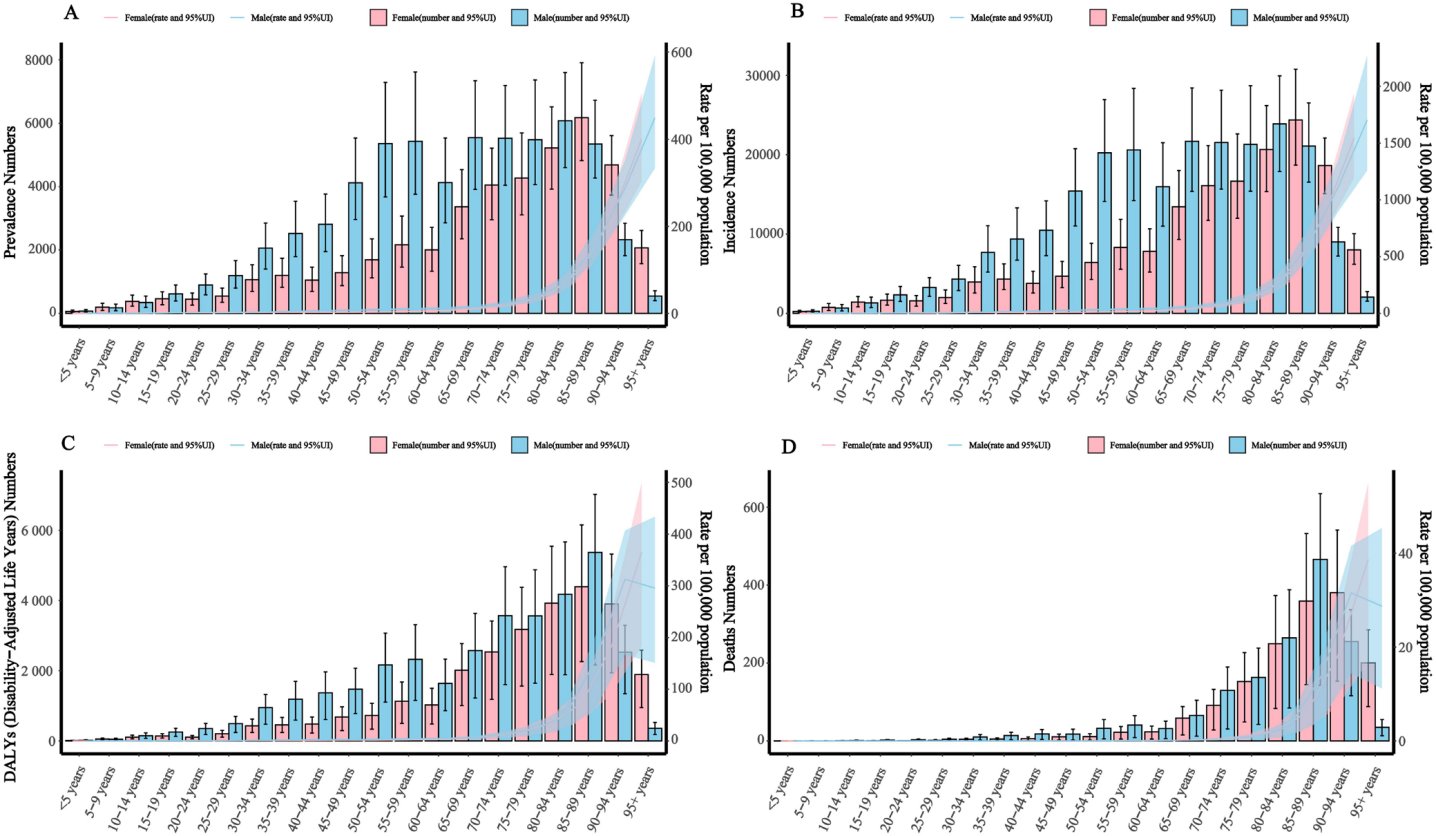
Sex‐ and age‐specific burden of pressure injuries in China in 2021. (A) Prevalent cases and prevalence rate; (B) Incident cases and incidence rate; (C) Disability‐adjusted life years (DALYs) and DALY rate; (D) Death cases and death rate. 95% uncertainty intervals (95% UI).

According to WHO data, China's population in 2021 was 1 428 242 112. Pressure injuries accounted for 102 938.9 prevalent cases (prevalence rate 7.2 [6.5–8.1] per 100 000; age‐standardised rate 5.9 [5.3–6.5]), 397 312.3 incident cases (incidence rate 31.9 [28.7–35.7]; age‐standardised rate 22.7 [20.7–25.1] per 100 000), 27 383.5 (14 016.7–36 289.8) DALYs (rate 4.4; age‐standardised rate injuries 3.7 per 100 000) and 3131.5 (1562.6–4126.8) deaths (crude and age‐standardised mortality both 0.2 per 100 000) (Table 1). By age, case numbers, incidence, DALYs and deaths peaked at 85–89 years, whereas all corresponding rates were highest in those aged ≥ 95 (Figure 1). By sex, men carried a higher burden than women from ages 50–89; women peaked in case numbers at 85–89. Prevalence and incidence rates peaked at ≥ 95 in both sexes. DALYs were higher in men before age 89 but higher in women thereafter, with a similar pattern for mortality (Figure 1).

**TABLE 1:** 1990 and 2021 China's burden of disease changes in pressure injuries.

	1990			2021			
	Number (95% UI)	Rate (95% UI)	ASR (95% UI)	Number (95% UI)	Rate (95% UI)	ASR (95% UI)	ASR EAPC (95% CI)
Prevalence							
Male	27 249.0 (23 705.9, 30 760.0)	4.5 (3.9, 5.1)	5.9 (5.3, 6.6)	60 557.4 (54281.8, 67 788.8)	8.3 (7.5, 9.3)	7.5 (6.7, 8.3)	1.2 (0.9, 1.4)
Female	15 591.4 (13 703.0, 17 626.2)	2.7 (2.4, 3.1)	3.6 (3.2, 4.0)	42 381.5 (38 053.8, 47 196.6)	6.1 (5.5, 6.8)	4.5 (4.1, 5.0)	1.1 (0.9, 1.4)
Both	42 840.4 (37 479.8, 48 387.9)	3.6 (3.2, 4.1)	4.7 (4.2, 5.3)	102 938.9 (92 962.8, 114 787.4)	7.2 (6.5, 8.1)	5.9 (5.3, 6.5)	1.1 (0.8, 1.3)
Incidence							
Male	104 094.3 (90 844.3, 117 001.9)	17.2 (15.0, 19.3)	22.9 (20.5, 25.7)	232 498.9 (209 054.3, 259 869.3)	31.9 (28.7, 35.7)	28.7 (26.0, 31.9)	1.1 (0.9, 1.4)
Female	59 414.3 (52 044.8, 67 267.0)	10.4 (9.1, 11.8)	13.8 (12.4, 15.4)	164 813.3 (148 298.8, 184 210.1)	23.7 (21.3, 26.5)	17.4 (15.8, 19.3)	1.1 (0.9, 1.3)
Both	163 508.7 (142 891.5, 184 055.1)	13.9 (12.1, 15.6)	18.3 (16.4, 20.5)	397 312.3 (360 058.4, 444 875.8)	27.9 (25.3, 31.3)	22.7 (20.7, 25.1)	1.1 (0.8, 1.3)
DALY							
Male	7222.3 (4607.3, 14 674.6)	1.2 (0.8, 2.4)	2.2 (1.4, 5.2)	34 550.4 (17 483.6, 45 040.7)	4.7 (2.4, 6.2)	4.7 (2.4, 6.1)	2.9 (1.7, 4.1)
Female	4742.8 (3045.7, 10 107.8)	0.8 (0.5, 1.8)	1.4 (0.9, 3.4)	27 383.5 (14 016.7, 36 289.8)	3.9 (2.0, 5.2)	2.9 (1.5, 3.8)	2.7 (1.4, 3.9)
Both	11 965.1 (7820.5, 21 703.8)	1.0 (0.7, 1.8)	1.7 (1.1, 3.5)	61 933.9 (36 313.3, 77 402.4)	4.4 (2.6, 5.4)	3.7 (2.2, 4.5)	2.8 (1.6, 4.0)
Deaths							
Male	117.7 (51.5, 466.0)	0.0 (0.0, 0.1)	0.1 (0.0, 0.3)	1553.8 (502.5, 2133.2)	0.2 (0.1, 0.3)	0.3 (0.1, 0.4)	3.9 (2.0, 5.7)
Female	124.8 (62.5, 450.3)	0.0 (0.0, 0.1)	0.1 (0.0, 0.2)	1577.7 (624.8, 2279.7)	0.2 (0.1, 0.3)	0.2 (0.1, 0.2)	3.4 (1.7, 5.2)
Both	242.5 (125.3, 702.8)	0.0 (0.0, 0.1)	0.1 (0.0, 0.2)	3131.5 (1562.6, 4126.8)	0.2 (0.1, 0.3)	0.2 (0.1, 0.3)	3.6 (1.9, 5.4)

### 3.2 Trends in the Disease Burden of Pressure Injuries in China From 1990 to 2021

From 1990 to 2021, the number of individuals with pressure injuries in China increased from 42 840.4 to 102 938.9 (EAPC = 3.1 [2.9, 3.3]); prevalence rose from 3.6 to 7.2 per 100 000 (EAPC = 2.5) age‐standardised prevalence increased from 4.7 to 5.9 per 100 000 (EAPC = 1.1). During the same period, incidence increased from 163 508.7 to 397 312.3 (EAPC = 3.2); the incidence rate rose from 13.9 to 27.9 per 100 000 (EAPC = 2.6) and the age‐standardised incidence rate from 18.3 to 22.7 per 100 000 (EAPC = 1.1). DALYs increased from 11 965.1 to 61 933.9 years (EAPC = 5.8); the DALY rate rose from 1.0 to 4.4 per 100 000 (EAPC = 5.2) and the age‐standardised DALY rate from 1.7 to 3.7 per 100 000 (EAPC = 2.8). Deaths due to pressure injuries rose from 242.5 to 3131.5 (EAPC = 9.1); both crude and age‐standardised death rates increased from near 0 to 0.2 per 100 000 (EAPC = 8.4 and 3.6, respectively).

By gender, both males and females showed a fluctuating upward trend in disease burden from 1990 to 2021, with males consistently higher, especially in incidence, DALYs and death. The gender gap in DALYs and age‐standardised death rates gradually widened over time.

**FIGURE 2:**
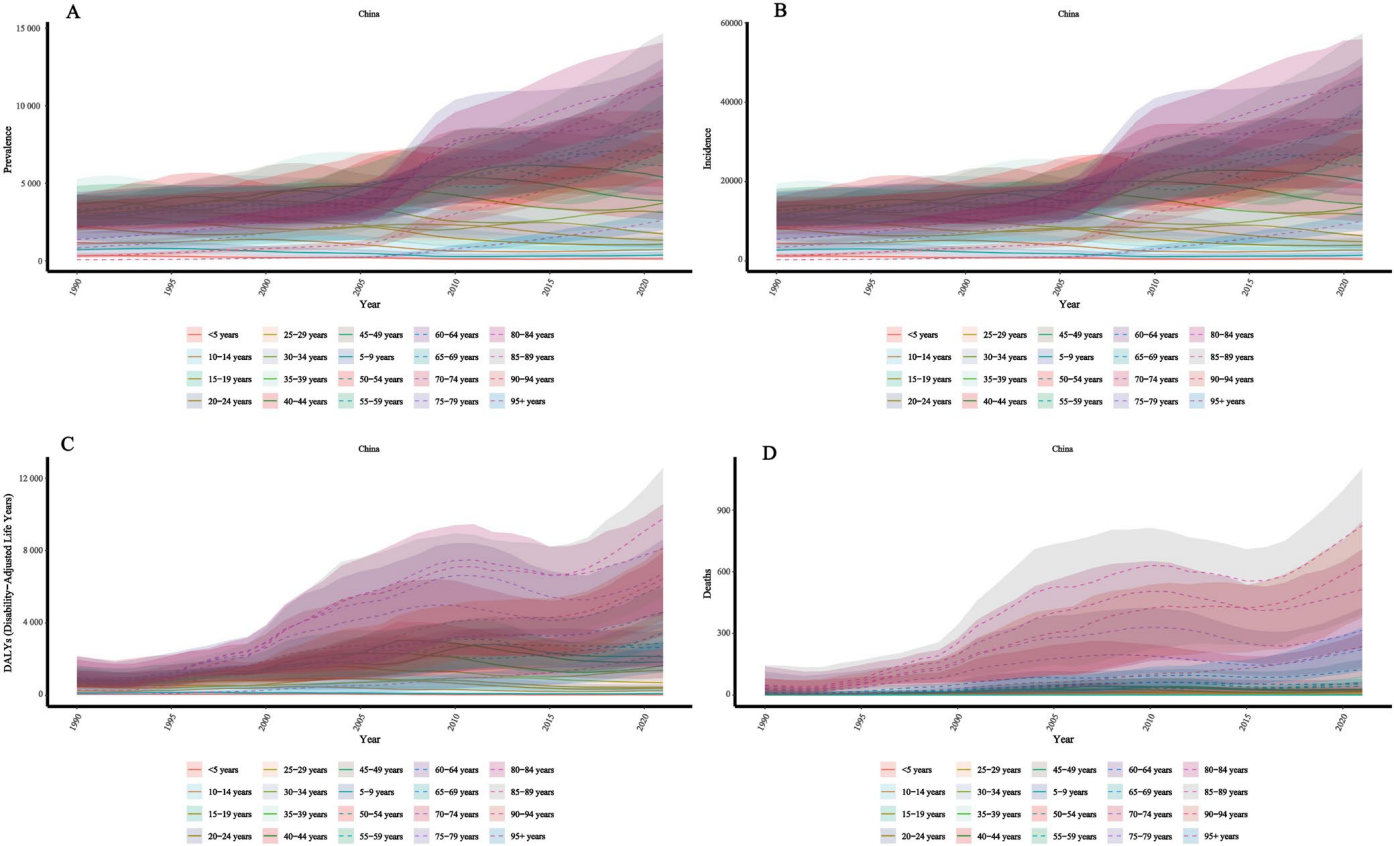
Age‐specific trends in the burden of pressure injuries in China from 1990 to 2021. (A) Prevalence rate; (B) Incidence rate; (C) Disability‐adjusted life year (DALY) rate; (D) Death rate. 95% uncertainty intervals (95% UI).

By age, prevalence and incidence rates increased across all age groups, with the highest in the 95+ group. DALY and death rates rose sharply between 1994 and 2004, then slightly declined, but overall continued to trend upward, peaking in those aged 95 and above (Figures 2 and S1).

### 3.3 Joinpoint Regression Analysis of the Disease Burden of Pressure Injuries in China From 1990 to 2021

**FIGURE 3:**
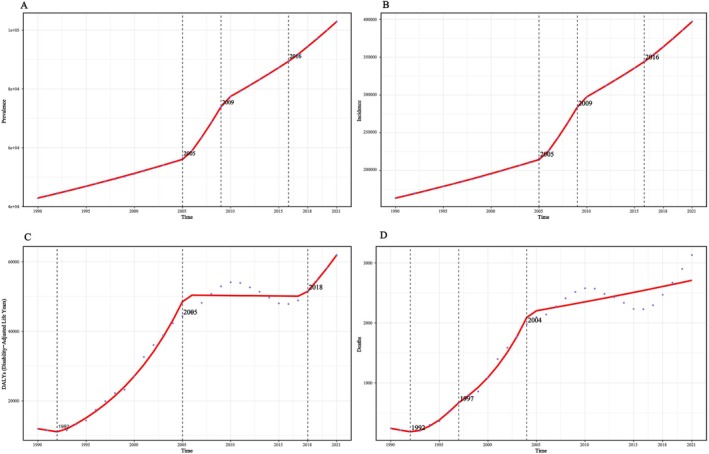
Joinpoint regression analysis of pressure injuries burden in China from 1990 to 2021. (A) Number of prevalent cases; (B) Number of incident cases; (C) Number of disability‐adjusted life years (DALYs); (D) Number of deaths.

The AAPC values for the number of prevalent cases, prevalence and age‐standardised prevalence were 1941.11%, 0.12% and 0.04%, respectively. For incident cases, incidence and age‐standardised incidence, the AAPC values were 7555.09%, 0.44% and 0.14%. For DALYs, DALY rate and age‐standardised DALY rate, the AAPC values were 1621.82%, 0.11% and 0.06%. For deaths, mortality and age‐standardised mortality, the AAPC values were 93.76%, 0.01% and 0.01%, respectively (Figures 3, S2 and S3).

### 3.4 Age‐Period‐Cohort Analysis of the Disease Burden of Pressure Injuries in China From 1990 to 2021

**FIGURE 4:**
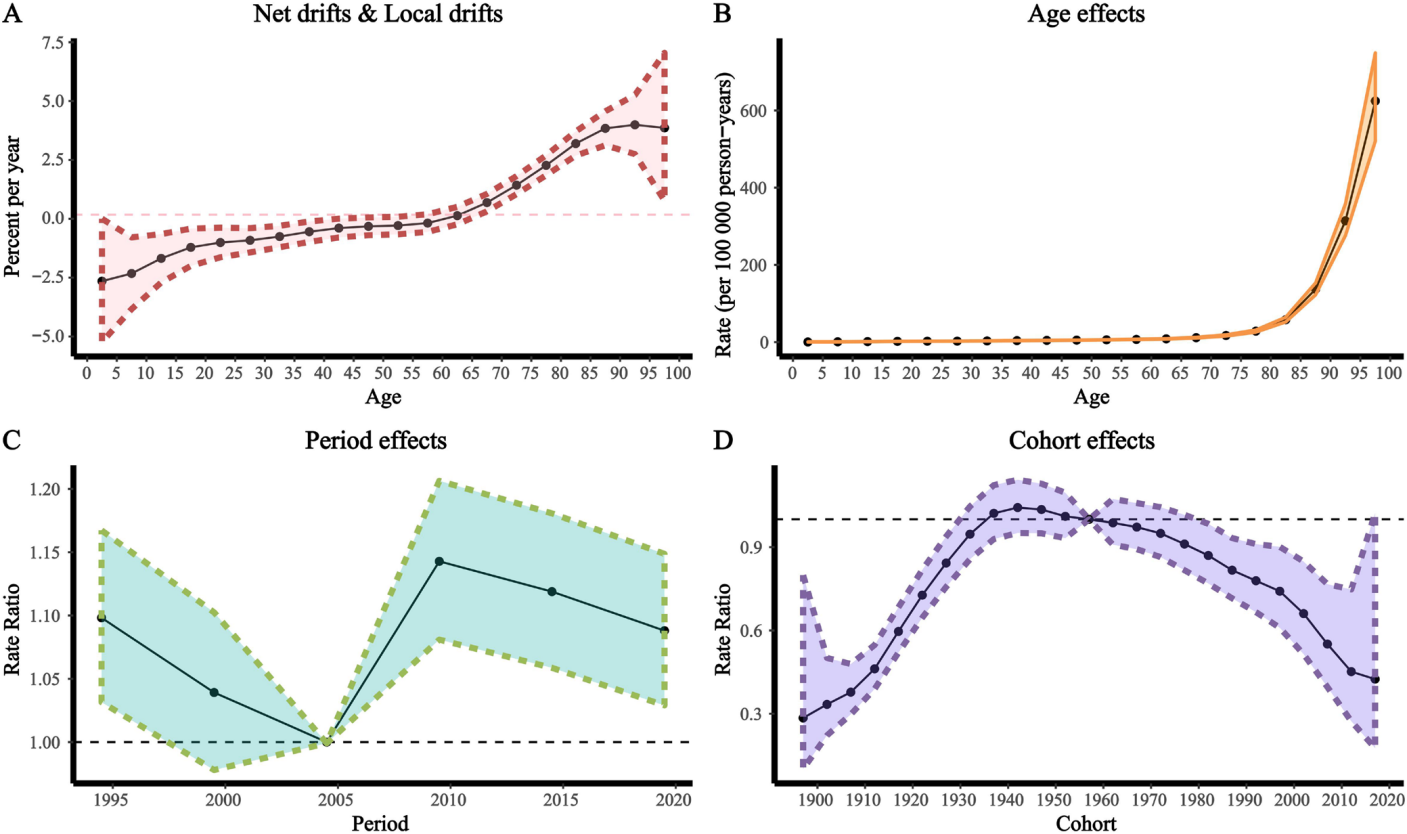
Age–Period–Cohort (APC) analysis of the prevalence burden of pressure injuries in China. (A) Net drift; (B) Age effect; (C) Period effect; (D) Cohort effect. 95% uncertainty intervals (95% UI).

From 1990 to 2021, the prevalence, incidence, DALY rate and mortality rate of pressure injuries in China showed varying degrees of increase, with net drift values of 0.17%, 0.13%, 1.78% and 4.19%, respectively. The age effect showed that all four indicators increased with age, reaching the highest levels in the 95+ age group. Using 2004 as the reference, the period effect showed a decrease in prevalence and incidence from 1990 to 2004, an increase from 2004 to 2009, peaking in 2009 (RR = 1.14), and then a decline. DALY and mortality rates continued to rise from 1990 to 2009, peaking in 2009 (RR = 1.04 for DALY, RR = 1.03 for mortality), followed by a decline. For the cohort effect, the highest risk of incidence was observed in the 1942 birth cohort (RR = 1.04–1.05), DALY risk peaked in 2002 (RR = 1.86) and mortality risk peaked in 2012 (RR = 8.19), with all showing a downward trend thereafter (Figures 4, S4–S6).

### 3.5 Decomposition Analysis of the Disease Burden of Pressure Injuries

To examine the effects of population aging, epidemiological changes and population growth on the burden of pressure injuries from 1990 to 2021, a decomposition analysis was conducted. The results showed that population aging was the main contributor to the increase in all burden indicators: 64.2% for prevalence, 65.0% for incidence, 46.5% for DALY rate and 49.1% for mortality rate (Figure S7).

### 3.6 Prediction of the Disease Burden of Pressure Injuries

**FIGURE 5:**
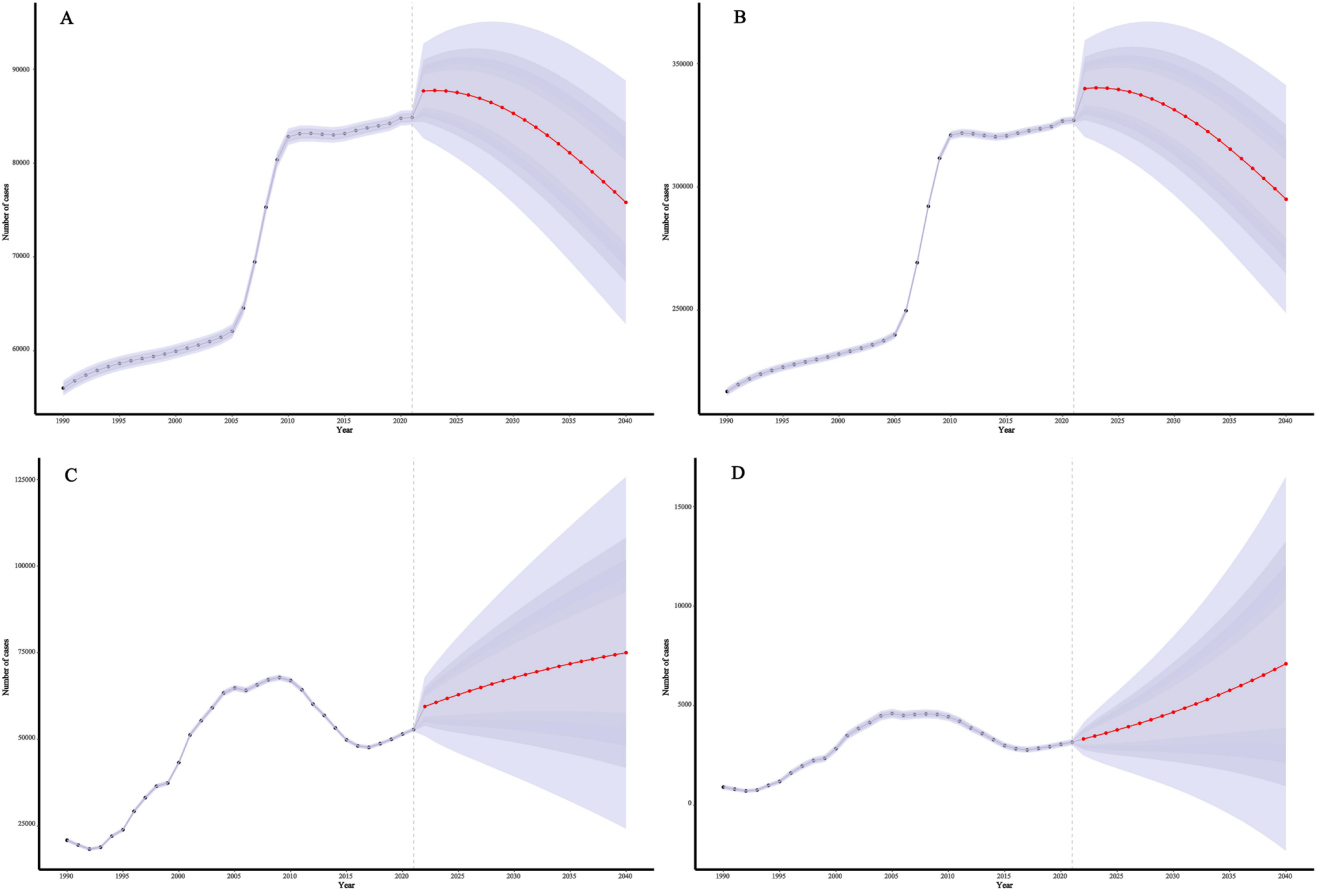
BAPC‐based projections of pressure injuries burden in China from 2022 to 2040. (A) Prevalent cases; (B) Incident cases; (C) Disability‐adjusted life years (DALYs); (D) Deaths.

Based on population projections from 2022 to 2040, this study used the Bayesian age‐period‐cohort (BAPC) model to estimate future trends in the burden of pressure injuries in China. The results showed that the age‐standardised prevalence and incidence rates are projected to decline, reaching 5.6 and 21.6 per 100 000 by 2040, respectively. The numbers of prevalent and incident cases are expected to decrease to 75 812.6 and 294 991. However, the number of cases among individuals aged 60 and above is projected to continue rising, with both prevalence and incidence rates increasing in the 85+ age group. In contrast, the burden of DALYs and deaths is expected to increase. The age‐standardised DALY rate and mortality rate are projected to reach 5.5 and 0.5 per 100 000, respectively, with total DALYs and deaths increasing to 75 019.4 and 7102.7. Most of this increase is expected to occur in middle‐aged and older populations. Overall, with continued population aging, older adults are projected to carry a greater share of the burden from pressure injuries in the future (Figures 5, S8–S16).

## 4 Discussion

The results of this study indicate that from 1990 to 2021, the age‐standardised rates of prevalence, incidence, DALYs and death from pressure injuries in China all increased continuously (EAPC > 0), reflecting a severe burden. Population aging was identified as the primary driver (contributing more than 46%). In addition, structured sex differences were evident (a higher burden among middle‐aged men, while mortality among women surpassed that of men in the oldest‐old), along with pronounced period and cohort effects. Projection analyses further suggest that China will face a paradoxical pattern in the future—declining age‐standardised rates but a sharp increase in the absolute number of cases among older adults. This contradiction implies that traditional prevention and control strategies are no longer sufficient to meet future challenges. There is an urgent need to shift the focus of pressure injury burden management toward the oldest‐old and end‐of‐life care settings, and to improve care quality through standardised protocols and technological innovation.

### 4.1 Pressure Injury Burden Dominated by Population Aging, With Structured Gender Differences and Marked Period and Cohort Effects

This study found that the disease burden of pressure injuries in China is characterised by population aging, structured gender differences and period and cohort effects. This result is consistent with previous research, which has identified age as one of the main risk factors for pressure injuries [16, 27, 28]. Decomposition analysis showed that 46.5%–65.0% of the increase in burden was attributable to aging, with the 85+ age group carrying a disproportionately high share. However, there is ongoing debate about the pathway through which aging affects pressure injuries. Some scholars suggest that aging is not a direct cause but acts through indirect factors such as reduced skin perfusion, decreased sensory function, limited mobility and impaired tissue repair [29, 30, 31, 32]. Nonetheless, the association between older age and higher risk of pressure injuries is clear. As China undergoes rapid aging, the burden among older adults requires attention. Compared with Japan, another aging country in Asia, China shows a similar prevalence of pressure injuries (7.2% vs. 7.7%–10%), but has fewer nursing personnel, with only 3.34 per 1000 people, which is much lower than Japan's standard [33]. This reflects a dual challenge in China: declining home‐based care and insufficient coverage of institutional eldercare.

In terms of gender, the study identified a distinct pattern: an overall higher burden in men, higher incidence in middle‐aged men and a reversal in very old women. Males had higher prevalence and incidence, possibly due to greater involvement in physically demanding and high‐risk occupations [34]. However, in the 85–89 age group, the burden among women surpassed that of men for the first time. This reversal has not been reported in previous studies and may be related to women's longer life expectancy (by 5.3 years) and higher rates of living alone [35, 36, 37, 38, 39, 40]. Current Chinese guidelines on pressure injuries do not include gender‐specific prevention strategies, indicating the need for further clinical and policy‐level attention.

The APC model revealed both period and cohort effects, reflecting the long‐term impact of historical and structural factors. The period effect showed an increase in burden between 2004 and 2009, with a peak in 2009 (prevalence RR = 1.14, incidence RR = 1.14, DALY rate RR = 1.04, mortality RR = 1.03), which may be linked to specific events during that time. The elevated risk among those born before 1942 (RR = 1.04–1.86) reflects cumulative damage from war and famine. The lower risk among cohorts born after 1960 (RR = 0.91) may be associated with policy reforms, such as the introduction of the household contract responsibility system in rural areas, which supported rural economic development and may have helped reduce disease burden.

These findings suggest that the prevention and treatment of pressure injuries are not only clinical issues but also relate to the equitable allocation of eldercare resources (e.g., improving access to eldercare institutions in rural areas) and targeted support for vulnerable groups (e.g., medical subsidies for survivors of war or famine), with the goal of building a healthcare system that better aligns with the needs of an aging population in China.

### 4.2 Inadequate End‐of‐Life Management of Pressure Injuries: Policy and Technological Breakthroughs Needed

The changes in pressure injury burden from 1990 to 2021 point to gaps in end‐of‐life management in China. Mortality increased at an average annual rate of 9.1% (95% CI: 7.3–10.9), much faster than the global rise over the same period [41, 42]. This pattern is linked to care for advanced pressure injuries. Infectious shock and multiple organ failure have become major causes of death. Many patients with late‐stage pressure injuries have severe comorbidities, limited mobility and disability [43, 44]. They are often transferred from tertiary hospitals to long‐term care or hospice facilities, where standardised wound care is replaced by more basic supportive treatment. Large regional gaps in health care also affect outcomes. In less developed areas, excessive antibiotic use is common in terminal cases, which raises the risk of drug‐resistant infections [45]. These findings suggest the need for routine infection surveillance in pressure injury care, and stronger enforcement of care standards in long‐term facilities, with graded and appropriate treatment.

Time‐trend results showed only a small rise in age‐standardised rates, but the absolute burden increased because of China's large population. From 1990 to 2021, prevalent cases rose by 141.0%, incident cases by 143.05%, DALYs by 417.65% and deaths by 1191.3%. This reflects a tension between clinical progress and rapid population aging. Although China has made policy efforts to improve outcomes in older adults, aging continues to outpace gains in care. For example, a study in Henan province showed that a three‐level linkage system, with structured interventions and staff training, improved pressure injury management in older patients [46]. Yet these improvements have not been enough to offset demographic change. The COVID‐19 pandemic further disrupted care. One study found that isolation measures and the use of protective equipment in tertiary hospitals added new challenges to wound management [47]. This highlights the need for flexible and innovative solutions during health crises. China should strengthen technological innovation to address the ‘technology–population’ gap, adopt prevention strategies tailored to its demographic structure, and include pressure injury prevention as a core indicator in the national Healthy Aging initiative.

### 4.3 Public Health Recommendations Under the ‘Overall Decline with Sharp Increase in the Oldest Age Group’ Scissor‐Like Burden Pattern

Based on model projections, the burden of pressure injuries in China is expected to follow a ‘declining total but increasing in the oldest age group’ pattern. By 2040, the total number of cases may decrease by 25.8% (from 397 312 to 294 991), while the burden among individuals aged 60 and above will increase, with the highest rates in those aged 85 and older. In response, China should develop a multilevel public health strategy tailored to an aging population. Drawing on Japan's experience as a super‐aged society, an integrated care system linking hospitals, communities and households should be established, with differentiated approaches for urban and rural settings. In cities, healthcare and elderly services should be better integrated; in rural areas, care can be delivered through village doctors, bedside services and mobile interventions [48]. Preventive measures should be context‐specific. This includes expanding the use of the Braden scale, app‐based risk alerts and skin monitoring and home‐based remote care plans [49]. Technological innovation should be encouraged and adapted to local conditions. Low‐cost, user‐friendly tools such as smart mattresses and pressure‐sensing patches can improve care in resource‐limited areas. AI‐assisted home robots and remote monitoring systems can help support older adults living alone, enabling real‐time alerts and targeted intervention. AI‐assisted home robots and remote monitoring systems can help support older adults living alone, enabling real‐time alerts and targeted intervention [50] In terms of payment reform, it is recommended to incorporate a risk adjustment factor for pressure injuries into the DRG system, considering age, risk scores and comorbidities. The US HACRP model may also offer a reference, linking incidence rates to reimbursement evaluation [51]. Including pressure injury prevention rates in community health service assessments could incentivise local institutions to actively participate in prevention efforts.

In addition, continuing education for healthcare workers should be strengthened. Public education materials—especially for rural and low‐literacy populations—should be clear, visual and easy to understand. Through the combined efforts of technological and policy innovation, care quality for older adults can be improved, providing a practical model for managing pressure injuries in aging societies both in China and globally.

## 5 Conclusion

This study, based on GBD 2021 data, reveals the spatiotemporal evolution patterns and multidimensional driving mechanisms of the disease burden of pressure injuries in China, providing scientific evidence and policy insights for managing pressure injuries in an aging society. The study shows that pressure injuries in China exhibit three main characteristics: dominance of aging, high prevalence among middle‐aged men and a reversal of gender differences in very old women, along with typical period and cohort effects. The predictive results indicate that by 2040, the total number of cases may decrease by 25.8%, but the incidence in the 85 and older group will increase, with deaths rising by 127%, forming a ‘scissors’ pattern. The study emphasizes the urgency of preventing and controlling pressure injuries in high‐risk and elderly populations, providing data support for addressing the burden of pressure injuries in the context of China's aging population and offering policy recommendations for pressure injury management in global aging societies. Preventing and managing pressure injuries is not only a clinical issue but also a touchstone for evaluating the effectiveness of the ‘Healthy China 2030’ strategy, making it a critical component of national aging governance and an evidence‐based model for other aging economies.

## 6 Limitations

Although this study provides a comprehensive assessment and forecast of the disease burden of pressure injuries in China, there are some limitations. First, this study primarily relies on the GBD database, which may have incomplete or outdated data on pressure injury burden in some low‐income or remote areas, potentially underestimating the disease burden in these regions. Second, because the GBD data are aggregate and are not linkable to individual primary diagnoses, we could not examine the proximate etiologies (e.g., specific chronic diseases or injury types) that lead to pressure injuries at the patient level. Future studies using patient‐level clinical or registry data are needed to characterise causal pathways and comorbidity‐specific risks. Third, the BAPC model used in this study for future burden predictions did not fully incorporate external variables such as policy changes, medical intervention advancements and other factors that may significantly impact future disease burdens, leading to some uncertainty in the predictions. Additionally, although this study considers major influencing factors like gender, age and population aging, the potential factors affecting the disease burden of pressure injuries are complex and diverse. Factors such as socioeconomic status, accessibility to medical resources, chronic comorbidities and the use of traditional Chinese medicine dressings were not fully incorporated, which may affect the comprehensive interpretation of the disease burden. Finally, as this study did not have access to detailed data from various provinces and cities in China, it could not conduct an in‐depth analysis of regional differences in the disease burden of pressure injuries. The disparities in economic development, healthcare resource allocation and health policies across regions may lead to significant differences in the burden of pressure injuries, which were not addressed in the study. In conclusion, these limitations suggest that future research should enhance the diversity of data sources, improve the variability of prediction models and better consider regional differences and potential influencing factors in the analysis.

## Ethics Statement

This study does not involve human ethical considerations.

## Consent

All authors reviewed the final manuscript and provided their approval for submission.

## Conflicts of Interest

The authors declare no conflicts of interest.

## Supporting information


**Figure S1:** Age‐specific trends in the burden of pressure ulcers injuries in China from 1990 to 2021.
(A) Number of prevalent cases; (B) Number of incident cases; (C) Number of Disability‐adjusted life years(DALYs); (D) Number of deaths.
**Figure S2:** Joinpoint regression analysis of rates for pressure ulcers injuries burden in China from 1992 to 2021.
(A) Prevalence rate; (B) Incidence rate; (C) Disability‐adjusted life year (DALY) rate; (D) Death rate.
**Figure S3:** Joinpoint regression analysis of age‐standardised rates for pressure ulcers injuries burden in China from 1992 to 2021.
(A) Age‐standardised prevalence rate (ASPR); (B) Age‐standardised incidence rate (ASIR); (C) Age‐standardised DALY rate; (D) Age‐standardised death rate (ASDR).
**Figure S4:** APC analysis of incidence burden of pressure ulcers injuries in China.
(A) Net drift; (B) Age effect; (C) Period effect; (D) Cohort effect.
**Figure S5:** APC analysis of DALY burden of pressure ulcers injuries in China.
(A) Net drift; (B) Age effect; (C) Period effect; (D) Cohort effect.
**Figure S6:** APC analysis of death burden of pressure ulcers injuries in China.
(A) Net drift; (B) Age effect; (C) Period effect; (D) Cohort effect.
**Figure S7:**. Decomposition analysis of pressure ulcers injuries burden in China.
**Figure S8:** BAPC‐based projections of age‐standardised burden of pressure ulcers injuries in China from 2022 to 2040.
(A) Age‐standardised prevalence rate (ASPR); (B) Age‐standardised incidence rate (ASIR); (C) Age‐standardised DALY rate; (D) Age‐standardised death rate (ASDR).
**Figure S9:** Age‐specific projections of prevalent cases of pressure ulcers injuries in China from 2022 to 2040.
**Figure S10:** Age‐specific projections of incident cases of pressure ulcers injuries in China from 2022 to 2040.
**Figure S11:** Age‐specific projections of DALYs of pressure ulcers injuries in China from 2022 to 2040.
**Figure S12:** Age‐specific projections of deaths due to pressure ulcers injuries in China from 2022 to 2040.
**Figure S13:** Age‐specific projections of prevalence rate of pressure ulcers injuries in China from 2022 to 2040.
**Figure S14:** Age‐specific projections of incidence rate of pressure ulcers inujuries in China from 2022 to 2040.
**Figure S15:** Age‐specific projections of DALY rate of pressure ulcers injuries in China from 2022 to 2040.
**Figure S16:** Age‐specific projections of death rate of pressure ulcers injuries in China from 2022 to 2040.

## Data Availability

The relevant data from the literature is stored in the attachments. For further inquiries, please feel free to contact the author.
