# Supplementary material for: Trends, Drivers and Projections of Pressure Injury Burden in China: Implications for National Healthcare Policy and Aging Governance
Source: Int Wound J. 2026 Jan 25;23(1):e70825. doi: 10.1111/iwj.70825 (PMC12832163; doi:10.1111/iwj.70825)

**1 Supplementary Materials**

This study employed the following statistical methods:

1. **Joinpoint regression analysis**

Joinpoint regression models were applied to examine the temporal trends in the burden of pressure ulcers in China from 1992 to 2021 . We used the Joinpoint Regression Program version 4.9.1.0, developed by the Statistical Research and Applications Branch of the National Cancer Institute, to estimate the annual percentage change (APC) and the average annual percentage change (AAPC), along with their respective 95% confidence intervals (CI) . The statistical significance of the trends was determined by testing whether the APC was significantly different from zero, using a significance level of P<0.05. An APC greater than 0 indicates an increasing trend in the burden of pressure ulcers, while an APC less than 0 indicates a decreasing trend. An APC equal to 0 reflects a stable trend over time.

1. **Age–period–cohort analysis**

The age-period-cohort (APC) model was employed to evaluate the influence of age, time period, and birth cohort on trends in the burden of pressure ulcers. Analyses were performed using the APC model online tool (https://analystools.cancer.gov/apc/). This model estimates temporal trends in prevalence, incidence, DALY rates, and mortality across different age groups, expressed as annual percentage change (APC).Relative risk (RR) was used to quantify the effects of age, period, and cohort. An RR greater than 1 indicates an increased relative risk of disease burden, whereas an RR less than 1 suggests a lower relative risk compared to the reference group.We also calculated the net drift, which refers to the linear time trend in pressure ulcer burden after accounting for age effects and the non-linear influences of period and cohort. It reflects the underlying direction and magnitude of change over time that cannot be solely attributed to population aging or specific generational or historical factors . Additionally, longitudinal age curves were plotted to illustrate changes in pressure ulcer burden across age groups over time. The net drift represents the overall APC for ASPR, ASIR, ASDR, and age-standardized death rate, capturing the logarithmic trend of the disease burden after adjusting for period and cohort effects .The statistical significance of the APC trends was assessed using the Wald χ² test. The APC model offers valuable insights into how age, period, and cohort factors contribute to the dynamic changes in the burden of pressure ulcers.In terms of data structure, the APC model divided the dataset into 20 consecutive five-year age groups (0–4, 5–9, …, 95+ years), covering the study period from 1992 to 2021. This resulted in six five-year periods (1992–1996, 1997–2001, …, 2017–2021) and 23 five-year birth cohorts (1907–1911, 1912–1916, …, 2017–2021). The model is based on a Poisson regression framework, and its general form is expressed as follows:

$$ln(Y_{abc})=\mu+\alpha_{a}+\beta_{b}+\gamma_{c}+\epsilon_{abc}$$

$ln(Y_{abc})$ denotes the natural logarithm of the burden of pressure ulcers in China;$\mu$ indicates the baseline risk level of the disease within a specific age group;$\alpha_{a}$ represents the age effect for the a-th age group;$\beta_{b}$ refers to the period effect for the b-th time period; $\gamma_{c}$ captures the cohort effect for the c-th birth cohort (where c = b – a + n, and n is the total number of age groups);$\epsilon_{abc}$ is the error term or residual.

**(3) Decomposition Analysis:** Decomposition analysis was used to assess the independent effects of population age structure, population growth, and epidemiological changes on the trends in pressure ulcers disease burden. By comparing ideal scenarios with actual results (controlling for other variables), the specific role of each factor in the trend was analyzed.

**(4) BAPC models**

This study incorporated the Bayesian Age-Period-Cohort (BAPC) model, which enhances the traditional APC framework by integrating Bayesian inference through Integrated Nested Laplace Approximation (INLA) . Unlike conventional APC models, the BAPC approach hierarchically incorporates baseline effects—such as demographic transitions or health policy changes—alongside age, period, and cohort components, effectively addressing issues like collinearity and overdispersion that commonly arise in long-term disease burden analyses.Specifically, the model employs Stochastic Partial Differential Equations (SPDEs) to approximate the posterior distributions of parameters. This enables stable model performance even with sparse or incomplete data and significantly reduces computational complexity .In this study, the BAPC model was used to forecast the burden of pressure ulcers in China from 2022 to 2040. The key modeling steps included : **Prior specification:** Weakly informative priors were assigned to age, period, and cohort effects to reduce potential bias stemming from subjective assumptions; **Posterior estimation:** INLA was used to efficiently compute posterior distributions of disease burden while accounting for uncertainties related to population dynamics and diagnostic variability; **Model validation:** The model’s goodness-of-fit was evaluated using the Deviance Information Criterion (DIC) and posterior predictive checks to ensure result reliability. This approach not only improves the accuracy of trend projections but also quantifies credible intervals for risk estimates, offering policymakers a probabilistic framework for designing targeted interventions across diverse age-cohort populations.

**2**  **Supplemental Figure Legends**

**Figure S1. Age-specific trends in the burden of pressure ulcers in China from 1990 to 2021**
(A) Number of prevalent cases; (B) Number of incident cases; (C) Number of Disability-adjusted life years(DALYs); (D) Number of deaths.


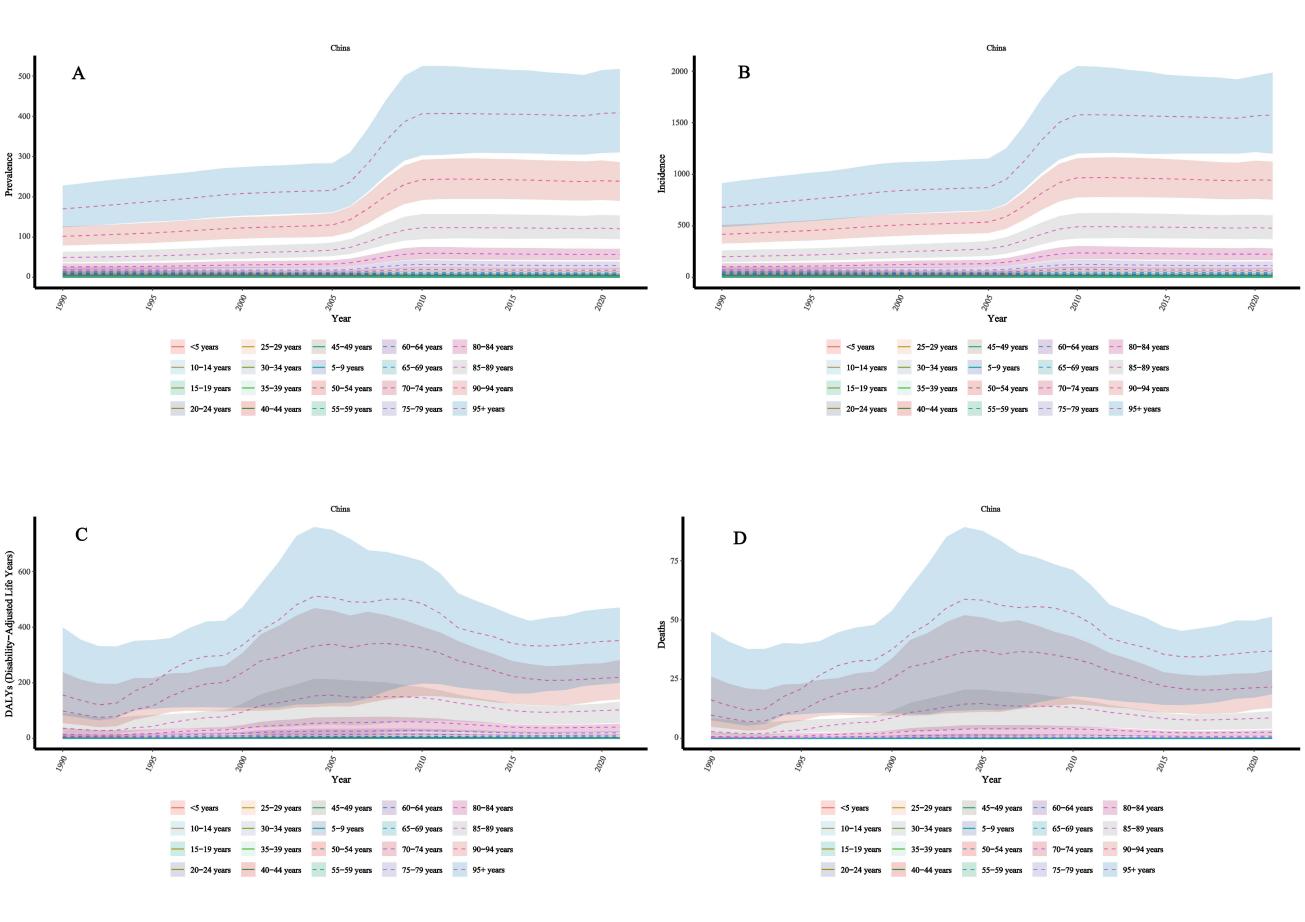


**Figure S2. Joinpoint regression analysis of rates for pressure ulcers burden in China from 1992 to 2021**
(A) Prevalence rate; (B) Incidence rate; (C) Disability-adjusted life year (DALY) rate; (D) Death rate.


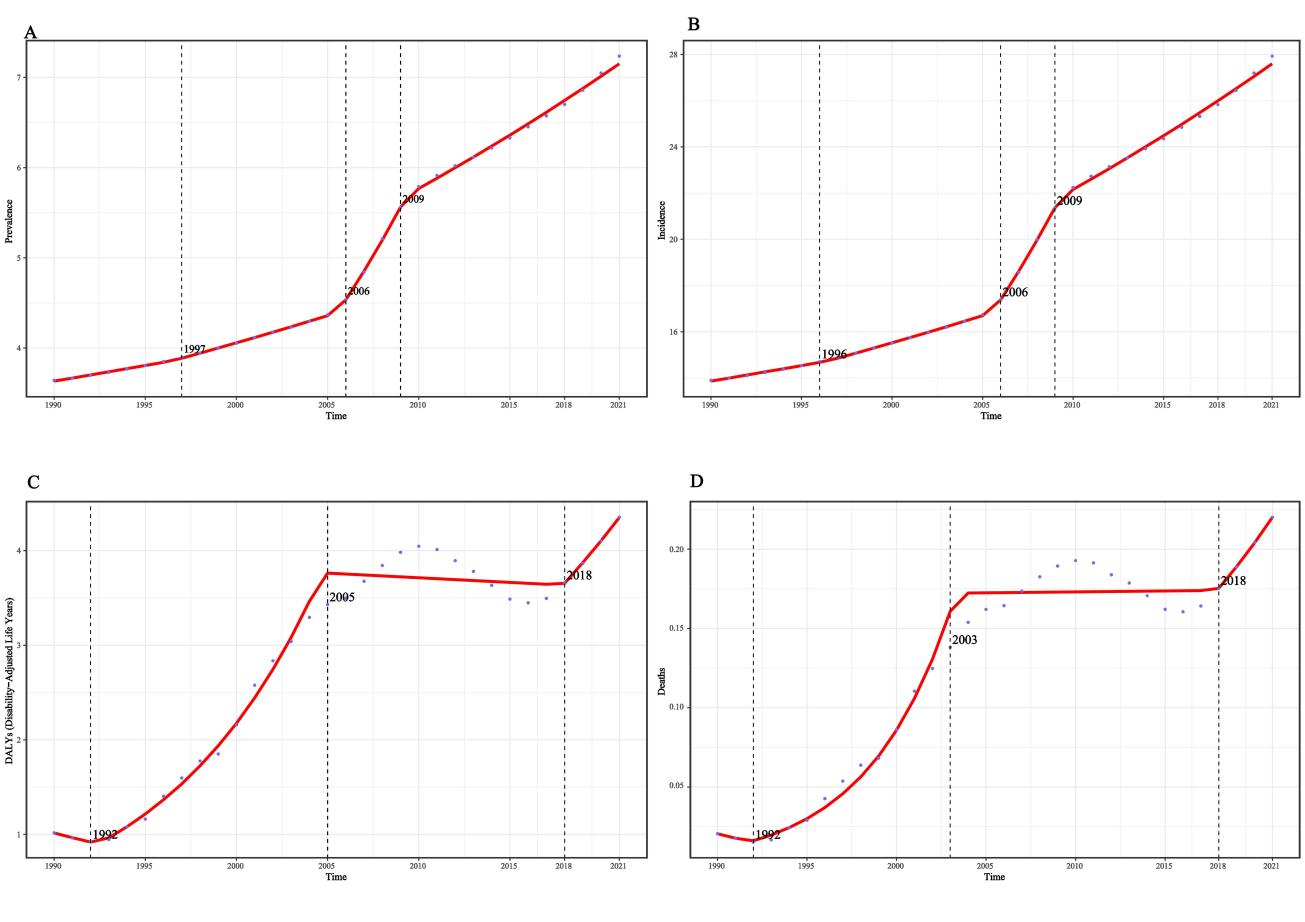


**Figure S3. Joinpoint regression analysis of age-standardized rates for pressure ulcers burden in China from 1992 to 2021**
(A) Age-standardized prevalence rate (ASPR; (B) Age-standardized incidence rate (ASIR); (C) Age-standardized DALY rate; (D) Age-standardized death rate (ASDR).


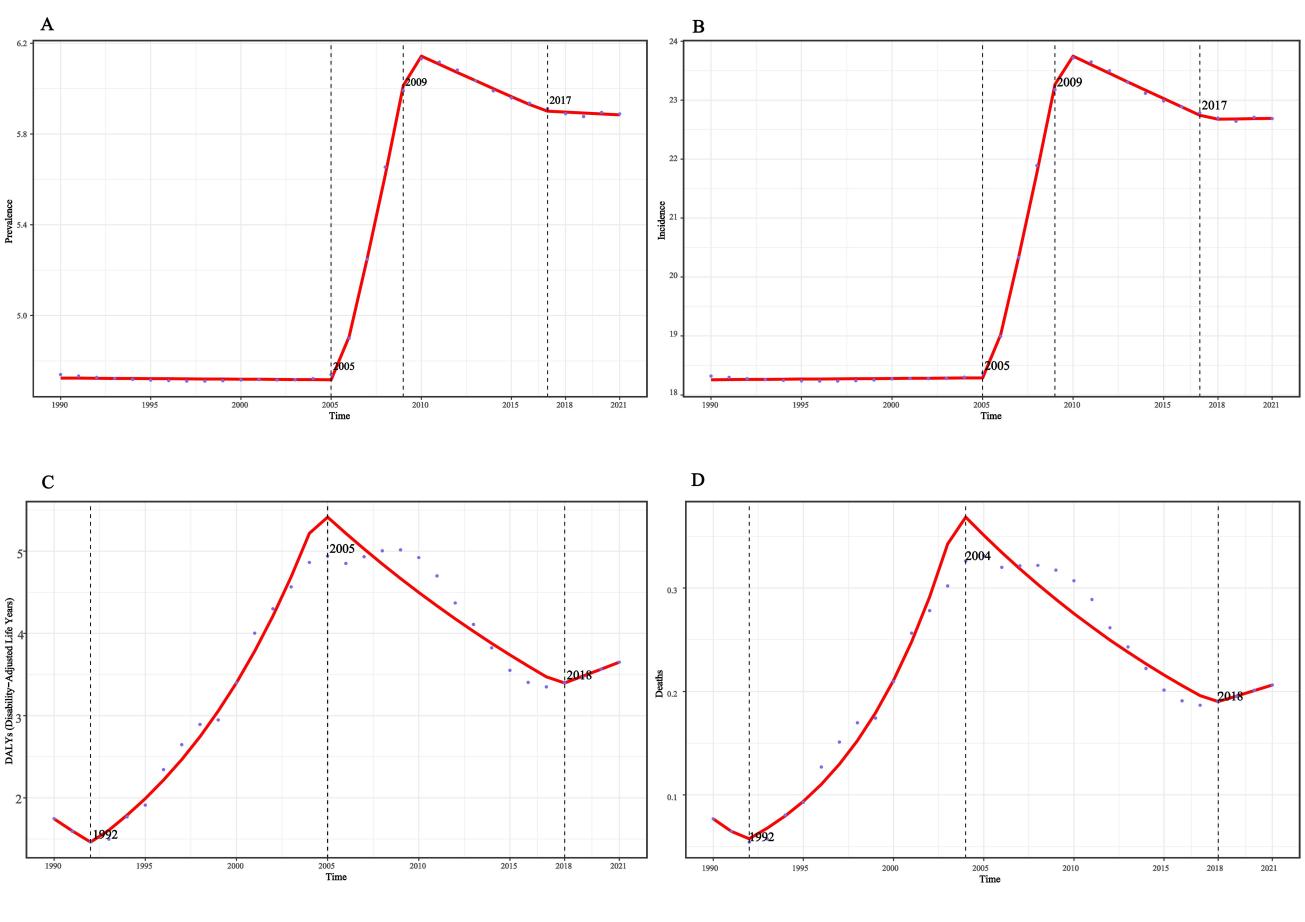


**Figure S4. APC analysis of incidence burden of pressure ulcers in China**
(A) Net drift; (B) Age effect; (C) Period effect; (D) Cohort effect.


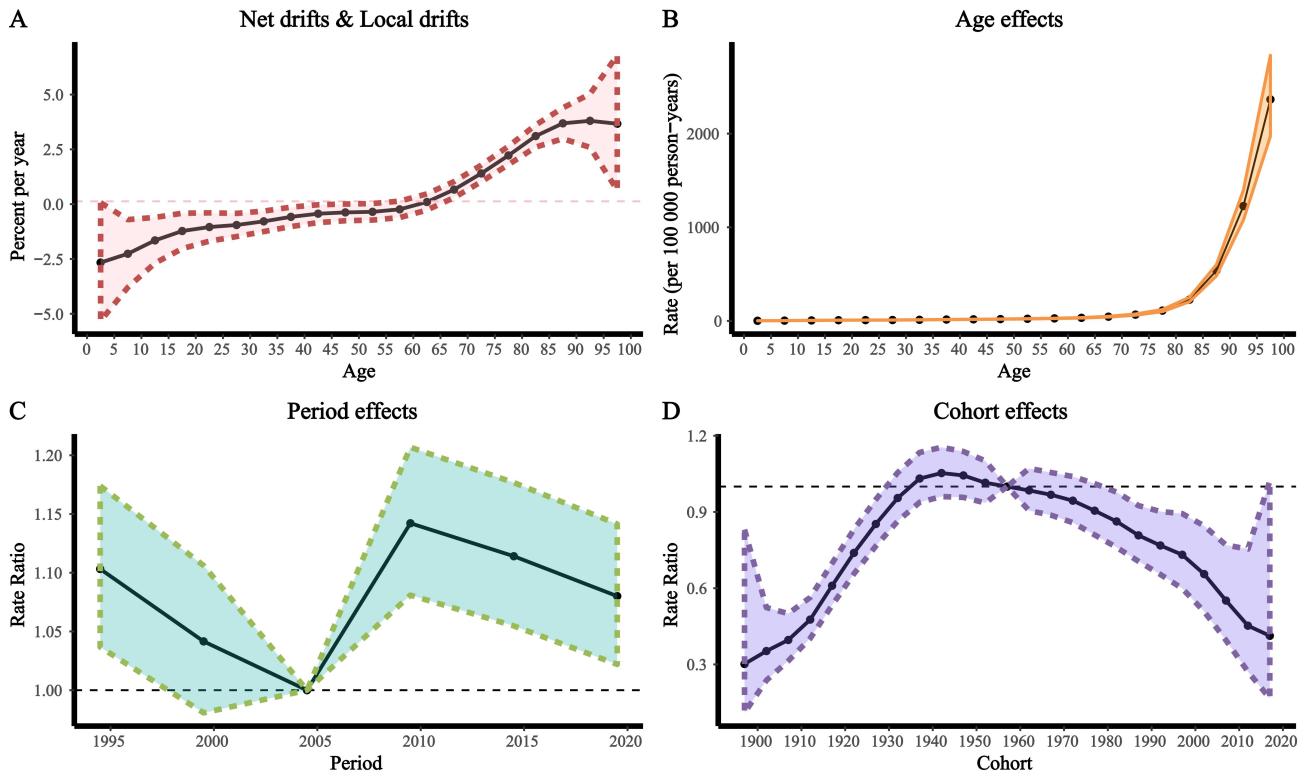


**Figure S5. APC analysis of DALY burden of pressure ulcers in China**
(A) Net drift; (B) Age effect; (C) Period effect; (D) Cohort effect.


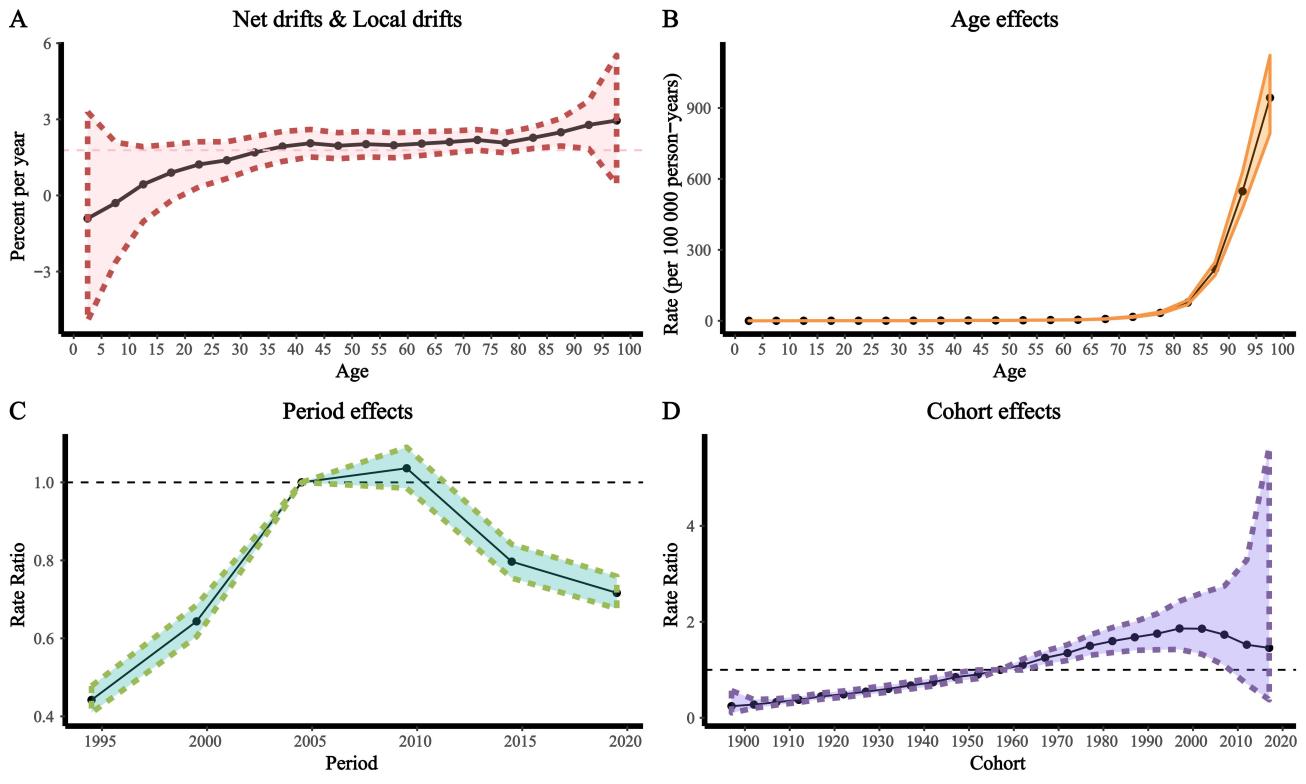


**Figure S6. APC analysis of death burden of pressure ulcers in China**
(A) Net drift; (B) Age effect; (C) Period effect; (D) Cohort effect.


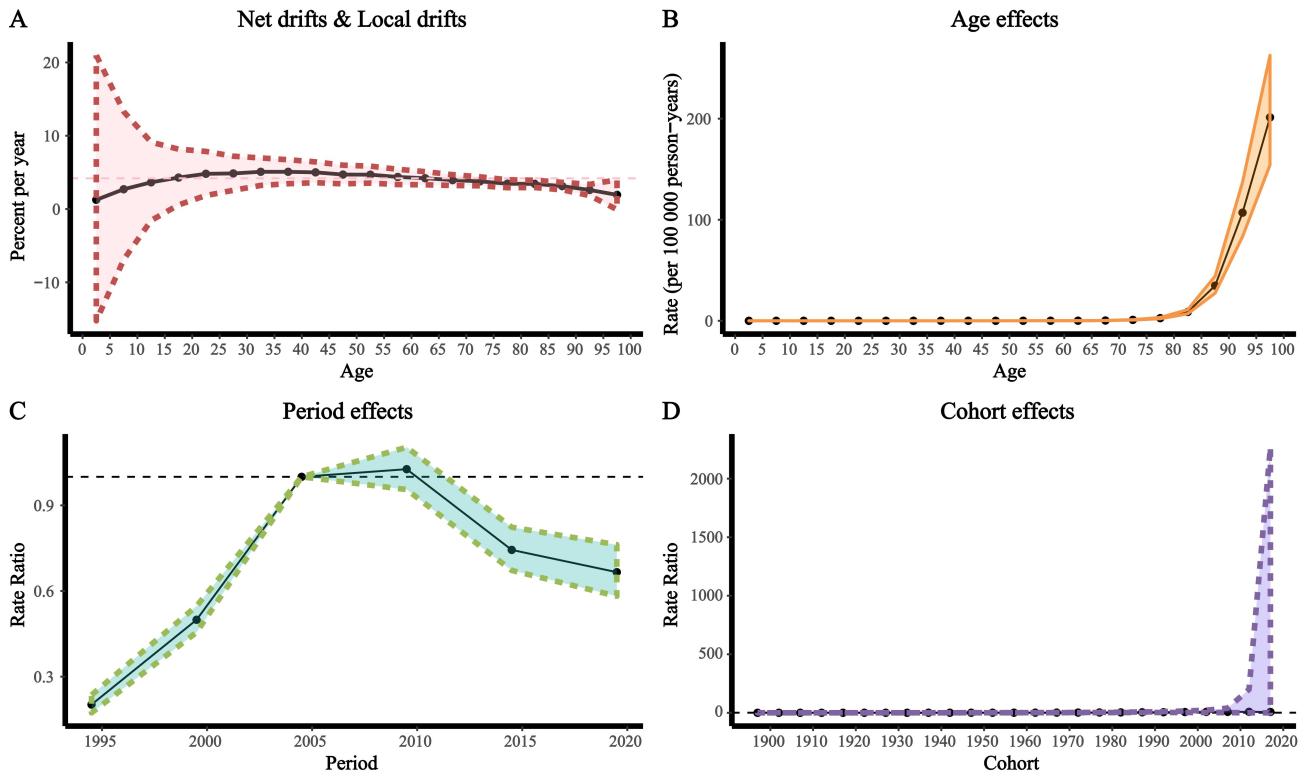


**Figure S7. Decomposition analysis of pressure ulcers burden in China**

(A) Prevalence burden; (B) Incidence burden; (C) Disability-adjusted life year (DALY) burden; (D) Death burden.


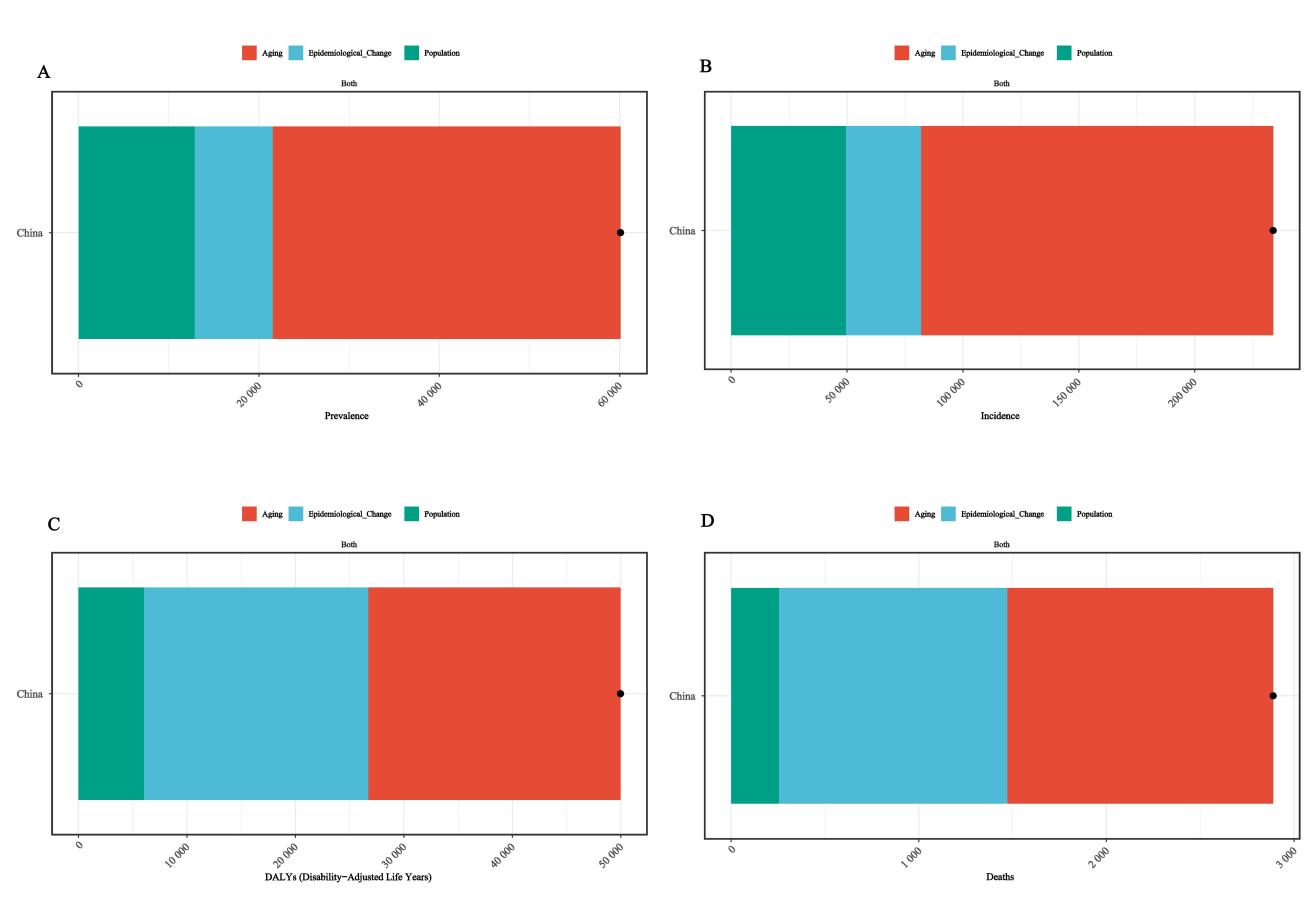


**Figure S8. BAPC-based projections of age-standardized burden of pressure ulcers in China from 2022 to 2040**
(A) Age-standardized prevalence rate (ASPR); (B) Age-standardized incidence rate (ASIR); (C) Age-standardized DALY rate; (D) Age-standardized death rate (ASDR).


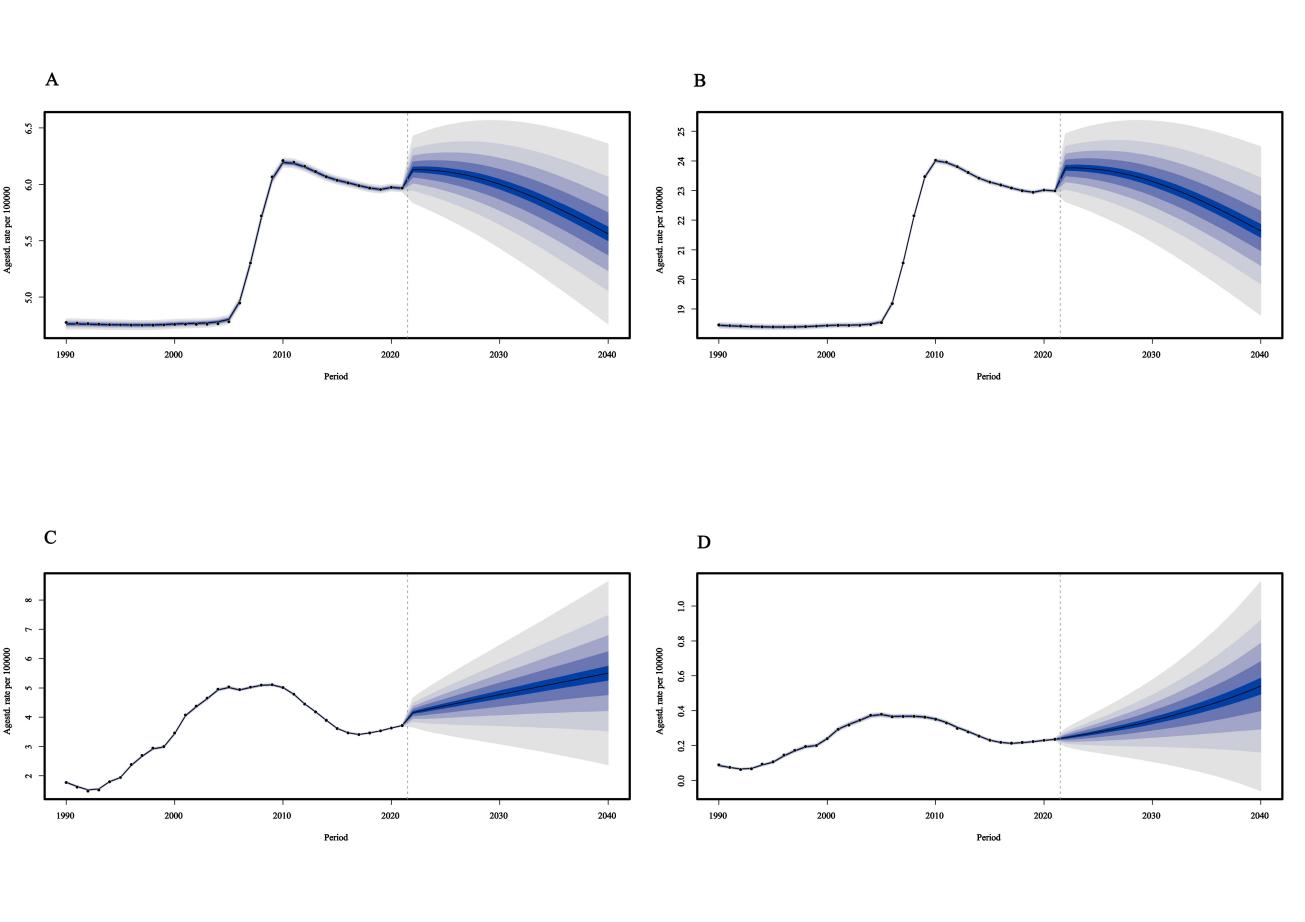


**Figure S9. Age-specific projections of prevalent cases of pressure ulcers in China from 2022 to 2040**


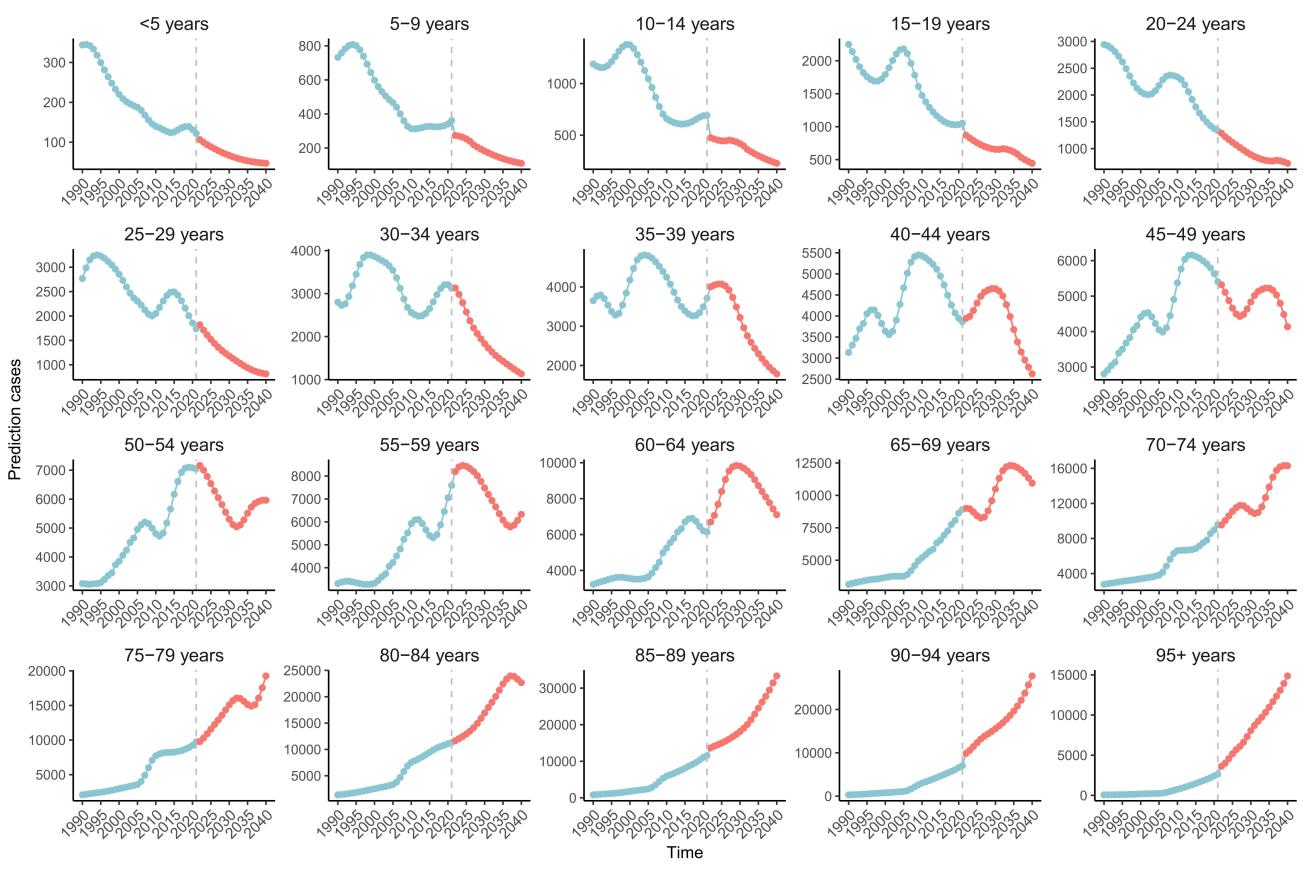


**Figure S10. Age-specific projections of incident cases of pressure ulcers in China from 2022 to 2040**


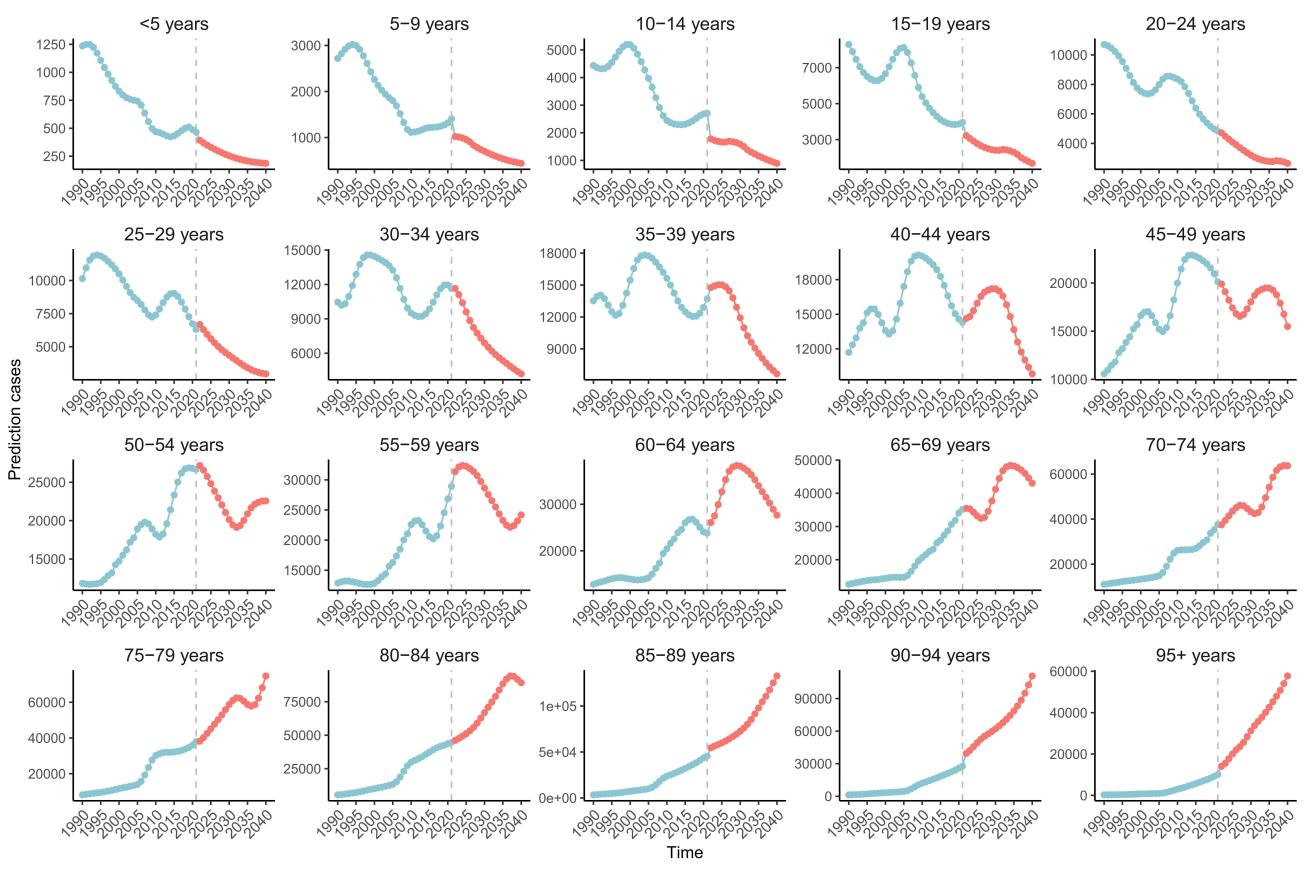


**Figure S11. Age-specific projections of DALYs of pressure ulcers in China from 2022 to 2040**


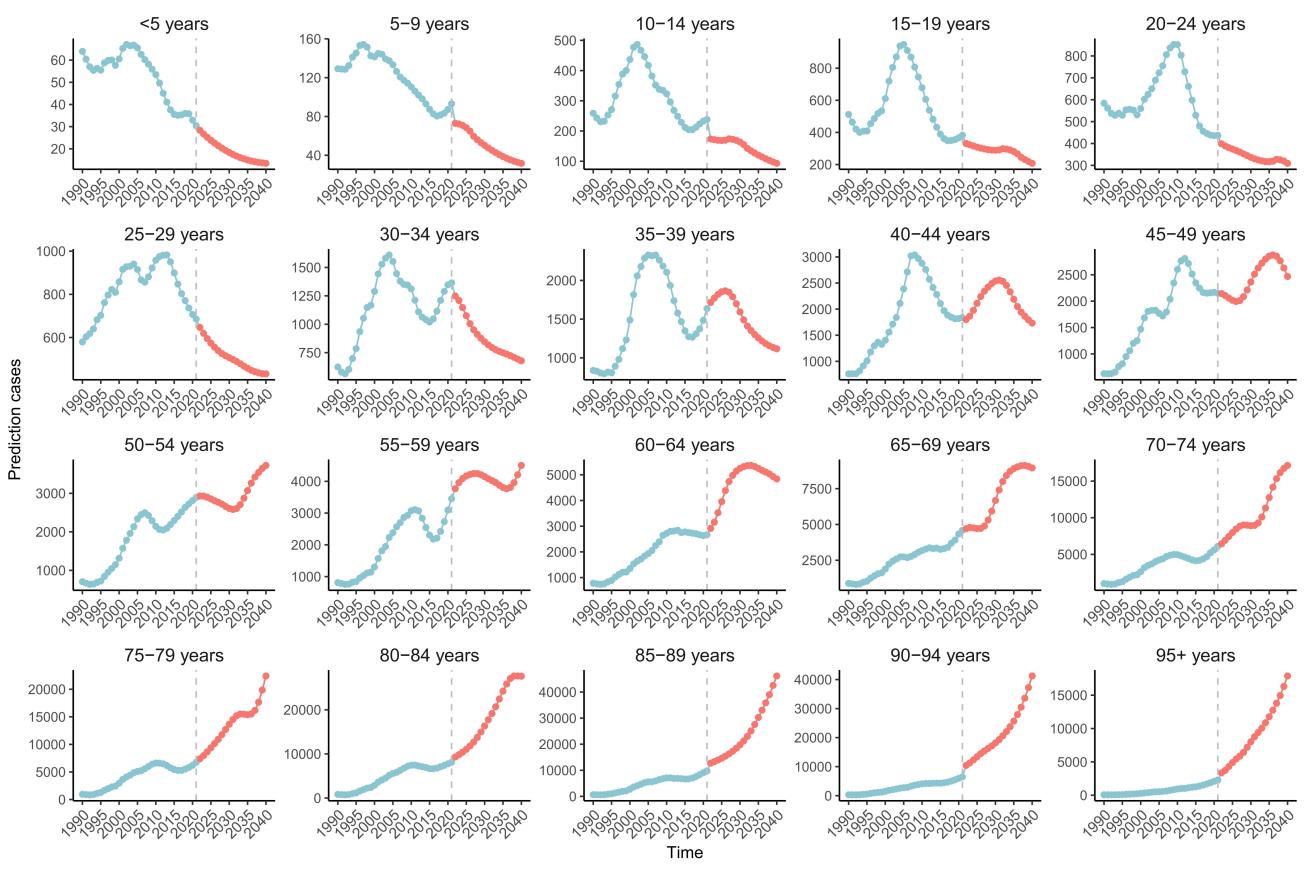


**Figure S12. Age-specific projections of deaths due to pressure ulcers in China from 2022 to 2040**


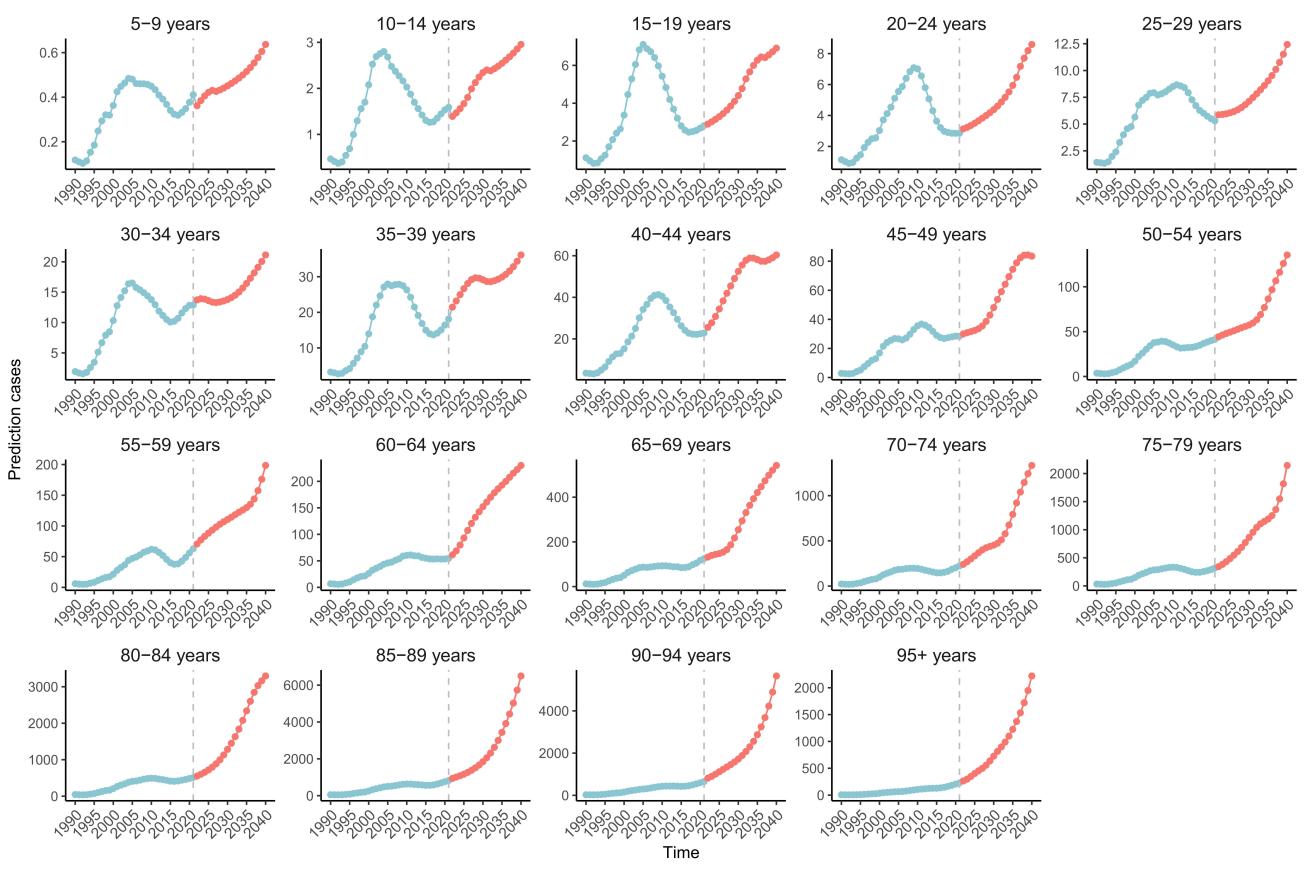


**Figure S13. Age-specific projections of prevalence rate of pressure ulcers in China from 2022 to 2040**


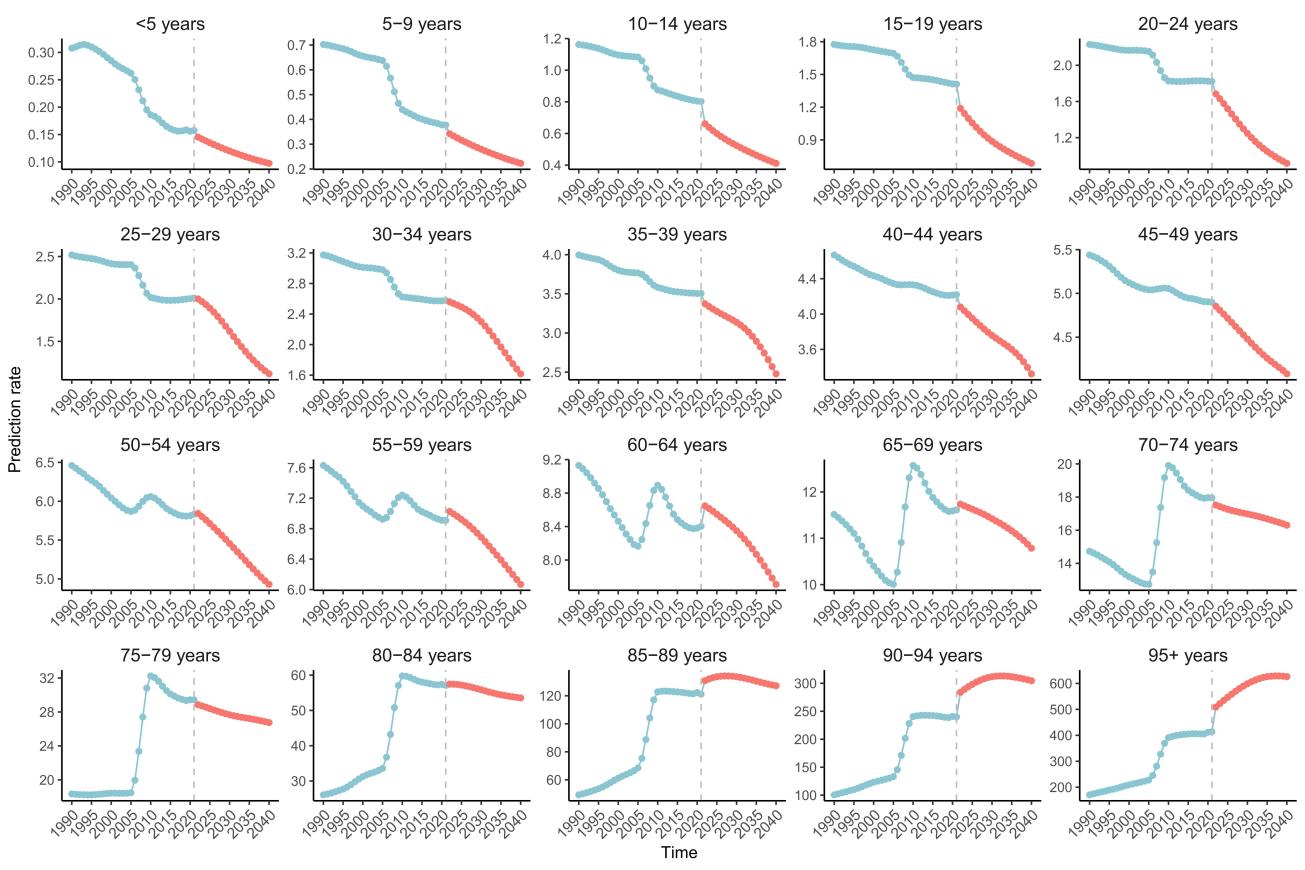


**Figure S14. Age-specific projections of incidence rate of pressure ulcers in China from 2022 to 2040**


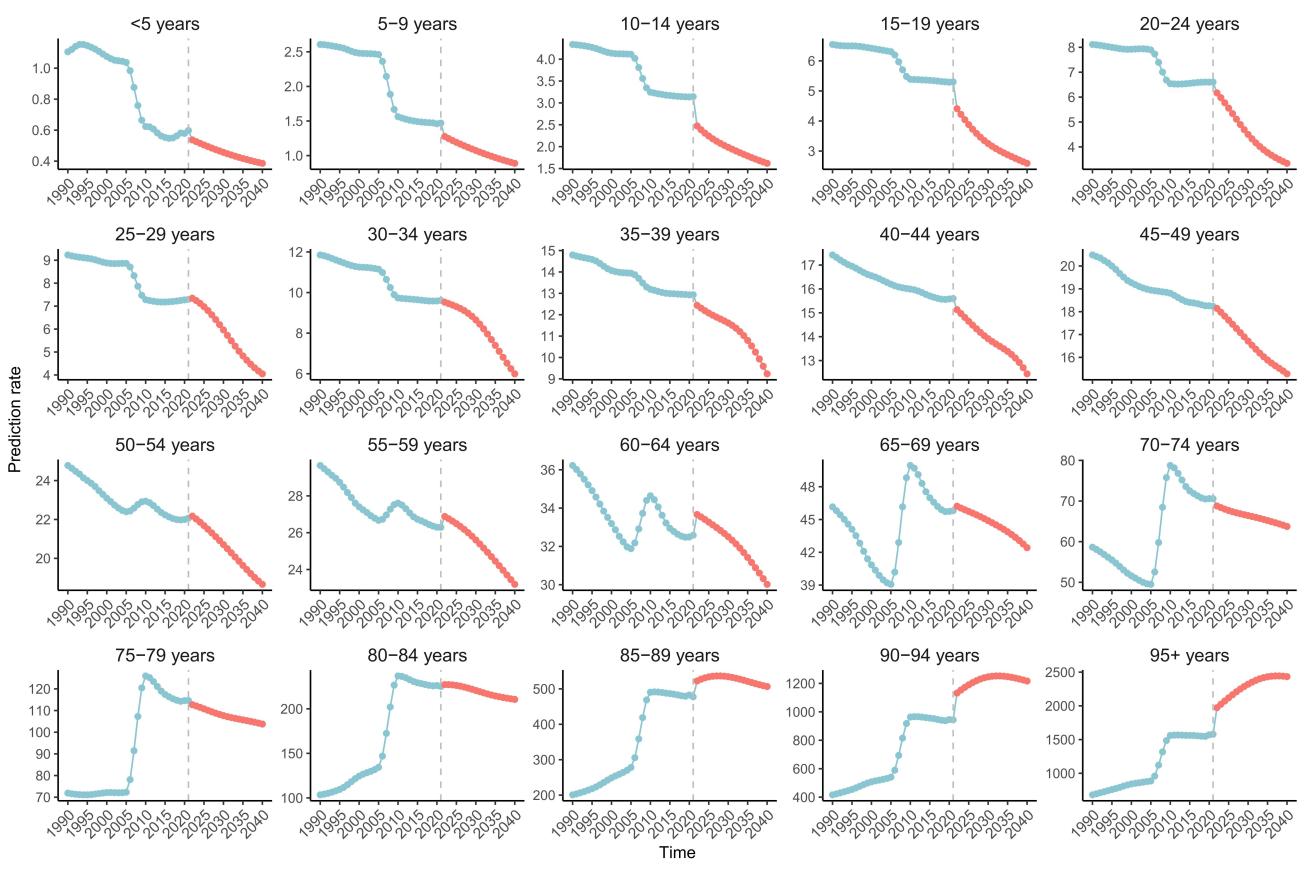


**Figure S15. Age-specific projections of DALY rate of pressure ulcers in China from 2022 to 2040**


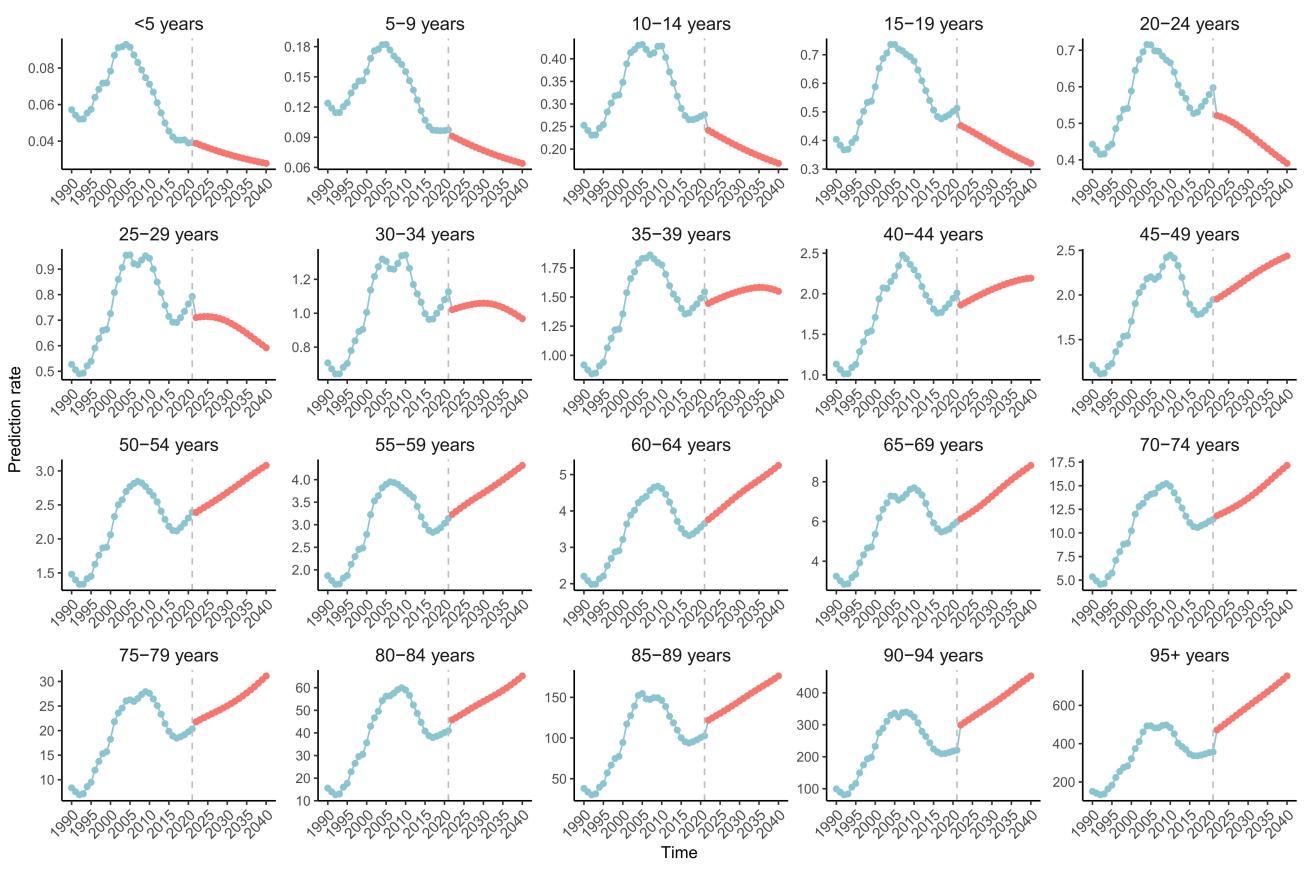


**Figure S16. Age-specific projections of death rate of pressure ulcers in China from 2022 to 2040**


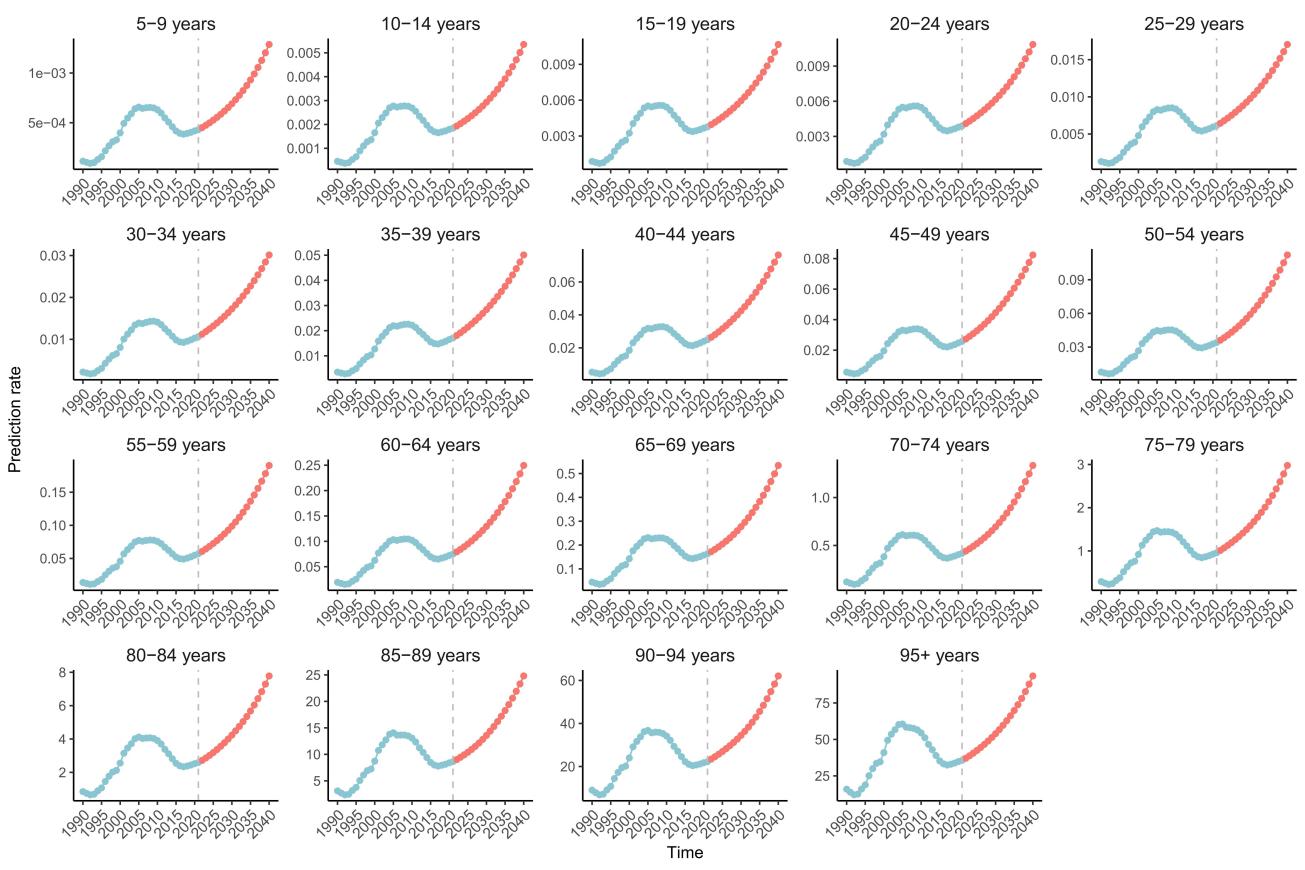

Supplement: Supplementary file 1 — Figure S1: Age‐specific trends in the burden of pressure ulcers injuries in China from 1990 to 2021. (A) Number of prevalent cases; (B) Number of incident cases; (C) Number of Disability‐adjusted life years(DALYs); (D) Number of deaths. Figure S2: Joinpoint regression analysis of rates for pressure ulcers injuries burden in China from 1992 to 2021. (A) Prevalence rate; (B) Incidence rate; (C) Disability‐adjusted life year (DALY) rate; (D) Death rate. Figure S3: Joinpoint regression analysis of age‐standardised rates for pressure ulcers injuries burden in China from 1992 to 2021. (A) Age‐standardised prevalence rate (ASPR); (B) Age‐standardised incidence rate (ASIR); (C) Age‐standardised DALY rate; (D) Age‐standardised death rate (ASDR). Figure S4: APC analysis of incidence burden of pressure ulcers injuries in China. (A) Net drift; (B) Age effect; (C) Period effect; (D) Cohort effect. Figure S5: APC analysis of DALY burden of pressure ulcers injuries in China. (A) Net drift; (B) Age effect; (C) Period effect; (D) Cohort effect. Figure S6: APC analysis of death burden of pressure ulcers injuries in China. (A) Net drift; (B) Age effect; (C) Period effect; (D) Cohort effect. Figure S7:. Decomposition analysis of pressure ulcers injuries burden in China. Figure S8: BAPC‐based projections of age‐standardised burden of pressure ulcers injuries in China from 2022 to 2040. (A) Age‐standardised prevalence rate (ASPR); (B) Age‐standardised incidence rate (ASIR); (C) Age‐standardised DALY rate; (D) Age‐standardised death rate (ASDR). Figure S9: Age‐specific projections of prevalent cases of pressure ulcers injuries in China from 2022 to 2040. Figure S10: Age‐specific projections of incident cases of pressure ulcers injuries in China from 2022 to 2040. Figure S11: Age‐specific projections of DALYs of pressure ulcers injuries in China from 2022 to 2040. Figure S12: Age‐specific projections of deaths due to pressure ulcers injuries in China from 2022 to 2040. Figure S13: Age‐spec [file IWJ-23-e70825-s001.docx]
